# DeepStrataAge: an interpretable deep-learning clock that reveals stage- and sex-divergent DNA methylation aging dynamics

**DOI:** 10.1038/s41514-026-00358-w

**Published:** 2026-03-13

**Authors:** Aaron Lin, Ilinca Giosan, Andrea Aparicio, Tao Guo, Max Melnikas, Laura Balagué-Dobón, Natàlia Carreras-Gallo, Sayf Al-Deen Hassouneh, Kirsten Seale, Alex Kowalewski, Brent Harrison, Ryan Smith, Lucas Paulo de Lima Camillo, Jessica Lasky-Su, Varun B. Dwaraka

**Affiliations:** 1 TruDiagnostic, 881 Corporate Drive, Lexington, KY USA; 2 University of Kentucky, College of Engineering, Department of Computer Science, Lexington, KY USA; 3 Channing Division of Network Medicine, Department of Medicine, Brigham and Women’s Hospital and Harvard Medical School, Boston, MA USA; 4 Shift Bioscience, Toronto, ON USA; 5 University of Cambridge, Cambridge, UK

**Keywords:** Biomarkers, Computational biology and bioinformatics, Genetics

## Abstract

Aging is the strongest risk factor for chronic diseases such as cardiovascular disease, Alzheimer’s, and cancer. DNA methylation (DNAm) clocks offer a promising measure of biological age, but most rely on linear models that miss non-linear dynamics and CpG interactions. To address this, we developed a deep neural network (DNN)-based DNAm clock trained on 29,167 samples profiled on Illumina EPIC v1.0 and v2.0 arrays. Using 12,234 CpGs selected through sex- and age-stratified correlations, our model achieved high accuracy (1.89 years) and outperformed published deep learning and elastic net based epigenetic clocks in a separate validation cohort. Using Shapley Additive Explanations (SHAP), we further uncovered phase-structured, wave-like dynamics in age-influential CpGs: an early-life module, a midlife transition, and late-life remodeling, with distinct timings by sex. These epigenetic waves cohere with non-linear, multi-omic “aging waves” reported in proteomics and longitudinal omics. SHAP further enabled interpretable CpG attribution, revealing structured, sex-specific aging phases: early-life male clocks involved developmental pathways, while female clocks emphasized cytoskeletal regulation; late-life divergence included immune activation in males and transcriptional remodeling in females. Our framework thus unites accuracy with mechanistic interpretability, revealing sex-specific windows when molecular aging reconfigures most rapidly.

## Introduction

Aging is the single greatest risk factor for the development of chronic diseases and mortality. Nearly every major age-related illness, such as cardiovascular disease, cancer, type 2 diabetes, Alzheimer’s disease, and osteoarthritis, shows a dramatic increase in incidence with advancing age. The risk of heart disease roughly doubles every decade after age 45, and the incidence of Alzheimer’s disease rises exponentially after age 65. The strong link between age and disease has sparked efforts to understand the biology of aging and to develop biomarkers that measure biological age, the functional state of cells and tissues, as distinct from chronological age, which is simply the number of years since birth. Although chronological age is a useful proxy for time, it does not capture the heterogeneity in aging trajectories across individuals. People of the same chronological age can vary substantially in physiological function, resilience, and susceptibility to age-related diseases. This disconnect has fueled interest in identifying markers of biological aging, measurable features that reflect the underlying rate or burden of aging at the molecular or systemic level. Biomarkers of aging are quantifiable biological traits that change with age and, ideally, capture core aspects of aging biology. An effective biomarker should not only correlate with chronological age but also predict clinically relevant outcomes, such as frailty, morbidity, and mortality.

Among molecular biomarkers of aging, DNA methylation (DNAm) has emerged as one of the most robust and informative candidates. DNAm involves the covalent addition of methyl groups to cytosine residues, primarily at cytosine-phosphate-guanine (CpG) dinucleotides, and plays a key role in the regulation of gene expression and genomic stability. Importantly, DNAm patterns exhibit highly reproducible, genome-wide alterations with age, reflecting both developmental programming and the accumulation of age-related molecular changes. These systematic, age-associated methylation shifts provide a quantitative signature that can be leveraged for age estimation. This observation has enabled the construction of epigenetic clocks-statistical models that use DNAm profiles to predict an individual’s chronological age or infer their biological aging rate. Furthermore, the same statistical pipeline has extended beyond just the estimation of age, to predict levels of protein, metabolite, and clinical states^1,2^, as well as overall risk of individual disease^3^.

The first generation of epigenetic clocks, developed by Hannum et al. and Horvath in 2013, demonstrated that DNAm profiles could accurately predict chronological age using a limited set of CpG sites. Horvath’s multi-tissue clock, based on 353 CpGs, was especially influential due to its applicability across a broad range of tissues. These models introduced the concept of epigenetic age, which correlates strongly with chronological age but offers limited insight into functional decline or disease susceptibility, as they were trained solely to match chronological time. To better capture age-related mortality and morbidity, second-generation clocks, such as *PhenoAge* and *GrimAge* integrated clinical and mortality data into their training. These models exhibited stronger associations with age-related outcomes, including morbidity, frailty, and lifespan, and have since become widely used in aging research due to their reproducibility and relevance to healthspan.

Despite recent advances, most DNAm clocks continue to rely on regularized linear models, particularly Elastic Net, which assume that each CpG contributes independently and linearly to age. This assumption fails to capture the non-linear methylation dynamics observed across the lifespan and ignores potential interactions among CpG sites, which may reflect shared regulatory networks or chromatin structure. Explicitly modeling such interactions is computationally infeasible due to the vast number of possible feature combinations. These limitations can impair model accuracy, particularly in tissues or age ranges that diverge from the training data. Moreover, theoretical models suggest that DNAm aging may follow non-linear, Gompertz-like trajectories, which are inherently difficult to approximate using linear frameworks. These challenges underscore the need for more flexible modeling strategies capable of capturing the complex, high-dimensional, and age-dependent structure of the methylome.

Deep neural networks (DNNs) provide a flexible framework for modeling the complex, non-linear relationships inherent in DNAm data. Unlike linear models, DNNs can capture high-order interactions and heterogeneous methylation patterns across tissues and life stages. Recent studies have demonstrated their utility: DeepMAge, a blood-based predictor, slightly outperformed Horvath’s clock and detected age-related disease signals, while AltumAge, trained on 142 datasets, improved accuracy across tissues and older age groups^4^. Other non-linear approaches have also shown promise. GP-age used Gaussian Process regression to achieve ~2.1 years median error with only 30 CpGs^5^, and cAge modeled non-linear CpG-age effects in over 24,000 samples using a modified Elastic Net framework^6^. These findings highlight the advantages of non-linear modeling for DNAm age prediction. However, many of these models, particularly DNNs, function as black boxes, making it difficult to interpret the contributions of individual CpG sites and limiting biological insight.

Furthermore, recent work has recognized aging as non-linear, with biomolecular systems undergoing punctuated reconfiguration at characteristic ages (“waves” of aging) in proteomes and multi-omic panels^7^. Therefore, we hypothesize that similar phase-structured dynamics exist in the methylome, and that leveraging an interpretable deep neural network and SHAP feature attributions across >20,000 blood methylome will allow us to identify coherent, sex-specific “epigenetic waves” that parallel reported proteomic and multi-omic inflection windows, while providing CpG-level resolution of the underlying regulatory programs.

To address the limitations of existing models, we present a DNN-based DNAm clock, named DeepStrataAge, that combines high predictive accuracy with interpretable outputs. The model is trained on 12,234 CpG sites from a cohort of 29,167 individuals taking a TruDiagnostic test using both EPICv1 and EPICv2 methylation arrays, thus capturing a broad and diverse methylation landscape. Compared to traditional linear models, DeepStrataAge achieves lower mean absolute error (MAE) and more effectively captures local non-linear aging dynamics, mitigating the systematic underestimation observed in older individuals. To enhance interpretability, we apply Shapley additive explanations (SHAP), a game-theoretic framework that quantifies the contribution of each feature to individual predictions. This enables the identification of CpG sites with the greatest influence on age estimation and the characterization of their effects across the lifespan. Our SHAP analysis revealed distinct, stage-specific methylation signatures. We observe that CpGs with high influence in early life are enriched for developmental processes, while those in later life are associated with inflammation, metabolic regulation, and cellular senescence. Notably, several CpGs displayed reversed methylation trajectories at advanced ages, suggesting potential compensatory mechanisms or age related epigenetic trajectories. By integrating deep learning with interpretable modeling, our approach offers a high-resolution view of DNAm aging, bridging the gap between predictive performance and mechanistic insight. This framework supports more nuanced applications of epigenetic clocks in aging research and precision health.

## Results

### Feature Selection and Lifespan Stratification of Epigenetic Aging

**Fig. 1:**
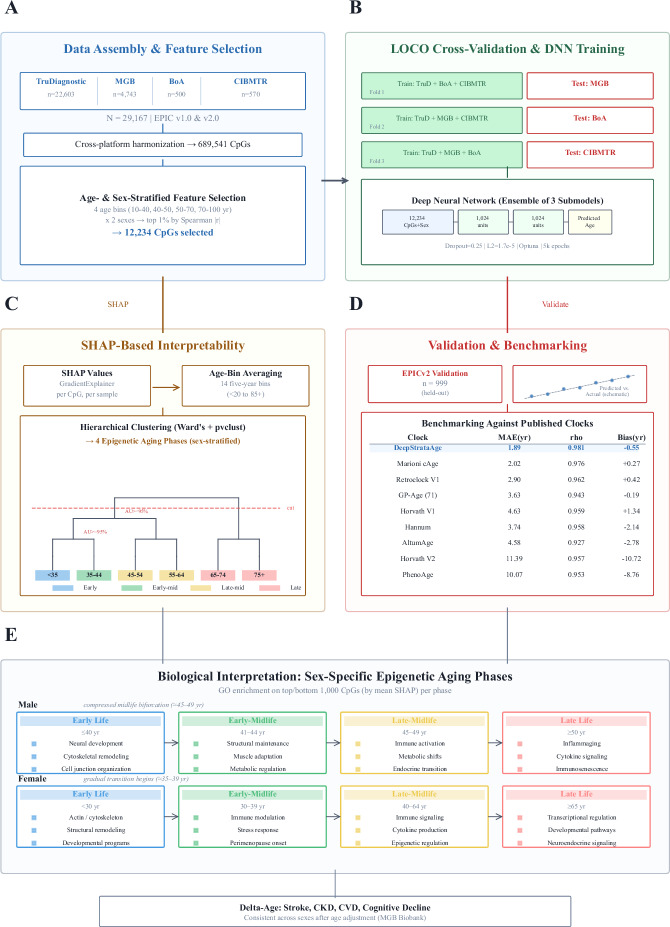
Study design and overview of the DeepStrataAge framework. This figure summarizes the end-to-end pipeline used to develop, validate, and interpret the DeepStrataAge model. Multi-cohort DNA methylation datasets were first harmonized to a shared CpG feature space and used for age- and sex-aware feature selection to define the final modeling input set (**A**). Model generalizability was then assessed using a leave-one-cohort-out (LOCO) strategy, where a deep neural network ensemble was trained on pooled cohorts and evaluated on a held-out cohort in each fold (**B**). The “1,024 units” label appears twice in (**B**) because the network schematic depicts two hidden layers of the same width (1024 units each). To move beyond prediction and characterize model behavior across the lifespan, SHAP-based attribution was used to summarize CpG contributions across age bins and to identify structured, sex-stratified epigenetic aging phases (**C**). Finally, performance was evaluated in an external held-out validation set and benchmarked against published epigenetic clocks (with the inset shown as a schematic illustration), and the resulting sex-specific phases were summarized with representative functional themes and linked to age-adjusted delta-age associations with age-related outcomes (**D**,**E**).

**Fig. 2:**
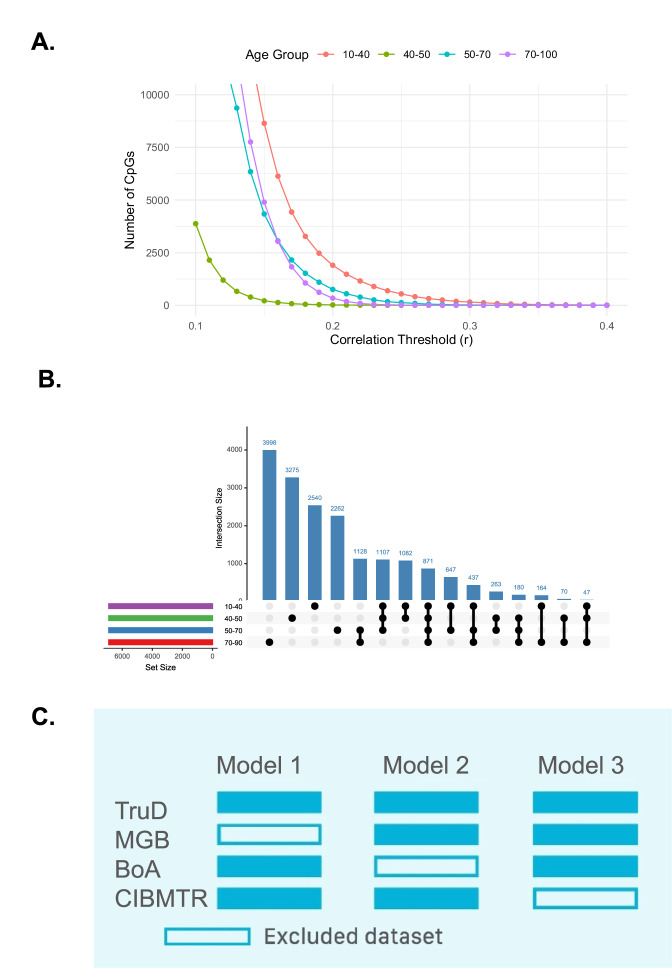
Feature selection and model training strategy. **A** Number of CpGs exceeding a given Spearman correlation threshold (r) with chronological age, stratified by age group (10–40, 40–50, 50–70, 70–100 years). Older age groups show a larger number of CpGs with stronger age correlations, indicating that age-associated methylation signals intensify in later life. **B** UpSet plot showing intersections of the top 1% most age-correlated CpGs across age groups. While many CpGs are unique to individual age bins, a subset is shared across multiple life stages, reflecting both conserved and age-specific methylation signatures. **C** Schematic of the leave-one-cohort-out (LOCO) cross-validation approach used to assess model generalizability. In each of three models, one external dataset is excluded from training and used solely for testing, while the remaining datasets serve as the training set.

To characterize sex-specific patterns of epigenetic aging across the lifespan, we developed a deep learning–based DNAm clock, DeepStrataAge, designed to balance predictive accuracy with biological interpretability. The model was trained on a harmonized dataset spanning multiple cohorts and platforms, incorporating feature selection stratified by age and sex. Supplementary Fig. 4 details the model architecture and Optuna-based hyperparameter optimization process, including the three LOCO-trained submodels used to generate ensemble predictions. Figure 1 provides an overview of the DeepStrataAge development pipeline, including feature selection, model training, hierarchical clustering for interpretability, and performance benchmarking on an EPICv2 validation dataset. The model outperformed existing clocks in both accuracy and correlation with chronological age. To identify age-informative CpG sites across the lifespan, we performed feature selection within four age strata (10–40, 40–50, 50–70, and 70–100 years), selecting CpGs based on their absolute Spearman correlation with chronological age. Figure 2A shows the relationship between correlation thresholds and the number of strongly age-associated CpGs across these strata. While all age groups exhibited a decay in CpG counts as the correlation threshold increased, older age groups (70-100 years) displayed a larger number of highly age-associated CpGs compared to younger groups, indicating that epigenetic aging signals become more pronounced in later life. We next examined the overlap of age-informative CpGs across life stages. Figure 2B shows shared and stage-specific CpG sets across the four age groups.Many CpGs were specific to individual life stages, highlighting stage-specific methylation signatures, while a subset was conserved across multiple age groups, suggesting a core epigenetic aging signature present throughout the lifespan. To assess whether age- and sex-stratification reveals CpGs not detectable in the full cohort, we compared Spearman and Pearson correlations within each subgroup. Supplementary Table 4 shows CpGs that become significant by Spearman only after stratification—despite being nonsignificant under Pearson—indicating that stratified rank-based analysis uncovers associations diluted in the unstratified dataset. Supplementary Table 5 lists CpGs that remain nonsignificant under both metrics, establishing a reference set that delineates features lacking detectable linear or monotonic age associations and clarifies which CpGs are uniquely uncovered through stratification. To contextualize the biological relevance of the age-informative CpGs identified across life stages, we annotated selected features with respect to CpG island context, genomic location, and known functional associations. Across age strata, selected CpGs were enriched in CpG shores and open sea regions relative to array background, consistent with prior reports that age-associated methylation changes preferentially occur outside promoter-proximal CpG islands. A substantial fraction of CpGs localized to gene bodies and distal regulatory elements, including enhancers, rather than canonical promoter regions. Several CpGs mapped to loci previously implicated in aging-related diseases and environmentally responsive epigenetic signatures, including sites associated with smoking and alcohol exposure in large-scale EWAS. Detailed annotations, including island context, nearest genes, and overlap with known exposure- and disease-associated CpGs, are provided in Supplementary Data 1. A summary of genomic context (gene feature and CpG island relationship) for these 12,234 selected CpGs is provided in Supplementary Fig. 7A, B to complement Supplementary Data 1. To evaluate model generalizability, we implemented a LOCO cross-validation framework (Fig. 2C). In this approach, one cohort (MGB, BoA, or CIBMTR) was systematically excluded for testing while the remaining cohorts and TruD were used for training. This strategy ensured robust evaluation of the model across diverse datasets.

**Fig. 3:**
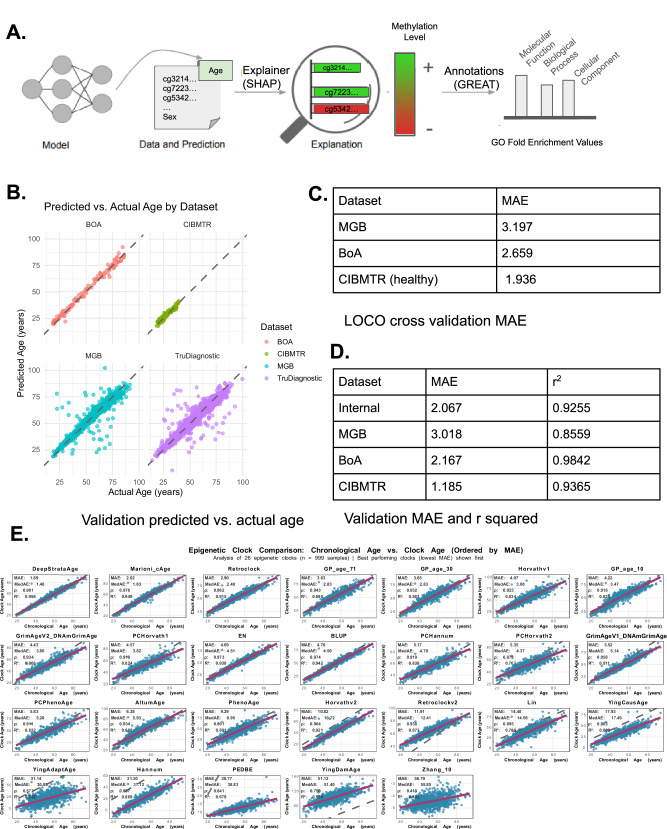
Model architecture, validation performance, and interpretability of the epigenetic aging predictor. **A** Overview of the modeling framework. A neural network was trained on DNA methylation profiles and sex to predict chronological age. SHAP (SHapley Additive exPlanations) values were used to identify CpGs most influential to predictions, which were then annotated via Gene Ontology (GO) enrichment to reveal biological pathways linked to epigenetic aging. **B** Predicted vs. actual age across four independent validation cohorts (BoA, CIBMTR, Internal, MGB), showing strong linear agreement and minimal deviation from the identity line. **C** Leave-one-cohort-out (LOCO) cross-validation mean absolute error (MAE) for MGB, BoA, and CIBMTR datasets, demonstrating generalizability across populations. **D** External validation results showing MAE and coefficient of determination (r²) for each cohort. Performance ranged from 1.19 to 3.02 years MAE with high r² (0.8559–0.9842). **E** Comparison of predicted vs. actual age across multiple aging clocks. Each scatter plot shows decimal chronological age (x-axis) vs. predicted age (y-axis), with MAE, RMSE, and Spearman ρ values annotated. Unique colors represent different clocks (legend in final panel); better performance is indicated by lower MAE/RMSE and higher ρ.

To build an interpretable model of epigenetic aging, we trained a deep neural network to predict chronological age from DNAm data and used SHAP values to identify CpG sites with the strongest influence on predictions (Fig. 3A). SHAP-based feature importance was then linked to Gene Ontology (GO) terms to uncover biological pathways underpinning age-associated methylation changes. Model performance was assessed using both LOCO cross-validation and independent validation datasets. As shown in Fig. 3B, predicted ages aligned closely with actual ages across cohorts, with minimal bias and tight clustering along the diagonal. LOCO and test-set MAEs ranged from 1.94 to 3.20 years across cohorts, with high r² values (0.8559–0.9842), indicating strong generalizability (Fig. 3C, D). In the independent TruDiagnostic EPICv2 validation cohort (n = 999), DeepStrataAge showed the best overall calibration among chronological age clocks (Fig. 3E, Table 1). It achieved the lowest MAE (1.89 years) and RMSE (2.79 years), with minimal bias (−0.55 years) and a regression slope of 0.948 and intercept of 2.15, indicating only mild compression of the age range around the identity line. Marioni_cAge and Retroclock also performed well (MAE 2.02 and 2.90 years; slopes 0.972 and 0.917; biases 0.27 and 0.42 years, respectively) but exhibited slightly larger error and/or greater deviation from the identity line. In contrast, several widely used clocks displayed substantial calibration drift despite high correlations; for example, Horvathv1 and Horvathv2 showed large positive or negative bias (1.34 and −10.72 years) and non-unit slopes (0.910 and 0.911), while outcome-based clocks, such as GrimAge and PhenoAge showed large negative offsets (e.g., PhenoAge bias −8.76 years) and slopes that diverged further from 1 (Table 1). These results indicate that DeepStrataAge most closely approximates a well-calibrated, near-identity mapping between DNAm age and chronological age in the held-out cohort.

**Table 1:** Calibration metrics for DeepStrataAge and comparator clocks in the held-out TruDiagnostic EPICv2 validation cohort (*n* = 999)

Rank	Clock	N	MAE	MedAE	RMSE	Bias	Pearson_r	Spearman_rho	R2	Slope	Intercept	Age_Accel_SD
1	DeepStrataAge	999	1.89	1.48	2.79	-0.55	0.978	0.981	0.956	0.948	2.15	2.73
2	Marioni_cAge	999	2.02	1.63	2.98	0.27	0.974	0.976	0.949	0.972	1.75	2.97
3	Retroclock	999	2.9	2.49	3.81	0.42	0.957	0.962	0.915	0.917	4.74	3.79
4	GP_age_71	999	3.63	2.83	4.88	-1.78	0.938	0.943	0.88	0.922	2.3	4.55
5	GP_age_30	999	3.65	2.83	4.88	-0.71	0.929	0.932	0.863	0.894	4.81	4.83
6	Horvathv1	999	4.07	3.08	5.58	1.34	0.913	0.923	0.834	0.91	6.03	5.42
7	GP_age_10	999	4.22	3.47	5.45	-0.2	0.91	0.918	0.828	0.878	6.16	5.45
8	GrimAgeV2_DNAmGrimAge	999	4.43	3.66	5.57	-2.5	0.932	0.934	0.868	0.757	10.17	4.98
9	PCHorvath1	999	4.57	3.82	5.84	-0.71	0.908	0.913	0.824	0.689	15.52	5.8
10	EN	999	4.69	4.51	5.52	-4.36	0.969	0.973	0.938	1.013	-5.02	3.39
11	BLUP	999	4.7	4.5	5.49	-4.51	0.971	0.974	0.942	0.956	-2.2	3.13
12	PCHannum	999	5.17	4.7	6.32	3.11	0.915	0.919	0.838	0.722	17.63	5.5
13	PCHorvath2	999	5.35	4.37	6.84	-1.42	0.876	0.878	0.767	0.612	18.82	6.7
14	GrimAgeV1_DNAmGrimAge	999	5.52	5.14	6.56	-4.99	0.954	0.958	0.911	0.784	6.3	4.26
15	PCPhenoAge	999	5.83	5.2	7.24	-4.69	0.907	0.911	0.822	0.858	2.75	5.52
16	AltumAge	999	6.38	5.93	7.72	5.88	0.926	0.934	0.858	0.924	9.85	5
17	PhenoAge	999	9.29	8.95	10.78	-8.76	0.895	0.901	0.801	0.965	-6.93	6.28
18	Horvathv2	999	10.82	10.72	11.33	-10.72	0.96	0.964	0.921	0.911	-6.05	3.66
19	Retroclockv2	999	11.91	12.41	12.93	11.74	0.934	0.938	0.873	0.675	28.71	5.41
20	Lin	999	14.48	14.56	15.77	-14.24	0.888	0.895	0.789	1.004	-14.43	6.77
21	YingCausAge	999	17.93	17.49	18.71	-17.79	0.896	0.903	0.802	0.823	-8.56	5.8
22	YingAdaptAge	999	31.14	30.88	33.2	-31.1	0.573	0.572	0.328	0.53	-6.55	11.63
23	Hannum	999	31.3	31.12	31.64	-31.29	0.932	0.937	0.869	0.842	-23.01	4.73
24	PEDBE	999	39.77	38.83	41.34	-39.77	0.824	0.841	0.679	0.14	5.16	11.28
25	YingDamAge	999	51.12	51.4	51.97	51.12	0.755	0.75	0.57	0.79	62.11	9.35
26	Zhang_10	999	56.7	55.85	58.14	-56.7	0.439	0.413	0.192	0.015	-5.25	12.84

Predicted age was regressed on chronological age; slope and intercept quantify deviation from the identity line, while bias (mean error), MAE, and RMSE summarize average and squared error. Age_Accel_SD is the standard deviation of age acceleration (predicted − chronological age).

To evaluate whether leukocyte composition could confound age prediction, we regressed DeepStrataAge and the nine other most age-correlated clocks in this dataset (Marioni_cAge, BLUP, EN, Horvathv2, Retroclock, GrimAgeV1_DNAmGrimAge, GP_age_71, Retroclockv2, Hannum) on 12 Houseman/EpiDISH fractions and compared raw versus cell-adjusted age associations (Supplementary Fig. 1). Leukocyte composition explained 26.3% of the variance in DeepStrataAge (R² = 0.263), comparable to peers across the Top-10 (R² range 0.240–0.321; Supplementary Fig. 1A). After adjustment, DeepStrataAge remained highly correlated with age (r = 0.846; Δr = −0.131 from r = 0.978), and the reduction was similar for comparators: Marioni_cAge r = 0.843 (R² = 0.252; Δr = −0.131), BLUP r = 0.841 (R² = 0.268; Δr = −0.130), EN r = 0.836 (R² = 0.263; Δr = −0.132), Horvathv2 r = 0.826 (R² = 0.240; Δr = −0.133), Retroclock r = 0.823 (R² = 0.255; Δr = −0.134), GrimAgeV1_DNAmGrimAge r = 0.823 (R² = 0.268; Δr = −0.131), GP_age_71 r = 0.805 (R² = 0.241; Δr = −0.133), Retroclockv2 r = 0.805 (R² = 0.321; Δr = −0.129), and Hannum r = 0.799 (R² = 0.315; Δr = −0.133) (Supplementary Fig. 1B). CD8 T-naïve cells were the dominant immune correlates across clocks, with DeepStrataAge showing r = −0.415 (Supplementary Fig. 1C). Together, these results suggest that while leukocyte composition explains a modest portion of variance, age associations remain strong after adjustment, supporting the biological robustness of DeepStrataAge.

### Hierarchical clustering reveals wave-like phases of epigenetic aging

**Fig. 4:**
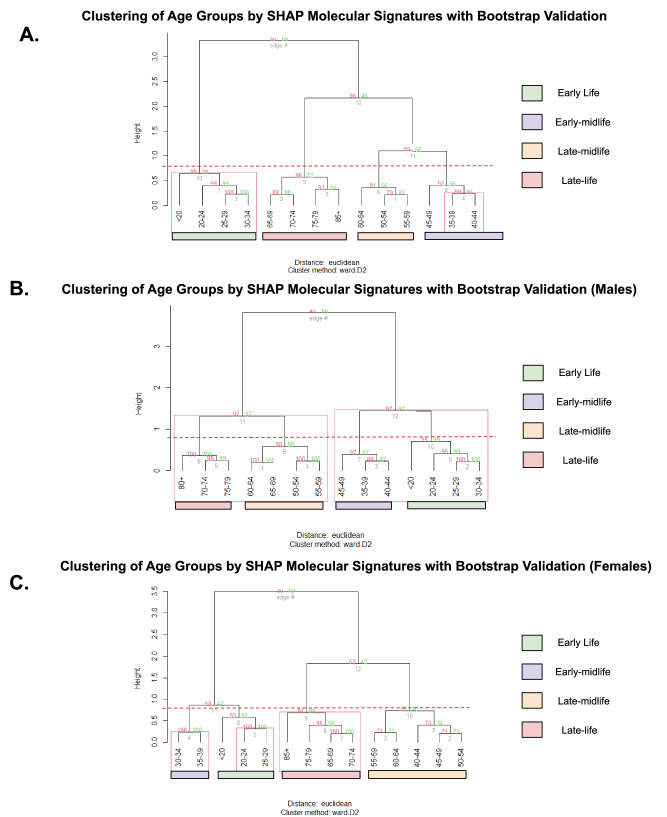
Hierarchical clustering of age groups by SHAP molecular signatures with bootstrap validation. Age groups (5-year bins; <20 and 85+ as open-ended) were clustered using Euclidean distance on SHAP-derived CpG influence profiles and Ward.D2 linkage. Multiscale bootstrap resampling (via pvclust) provides approximately unbiased (AU, red) and bootstrap probabilities (BP, green) above each dendrogram edge; AU ≥ 95% indicates strong support. Colored bars beneath the leaves reflect the four-phase schema used for interpretability (legend at right). The red dashed line in (**A**) marks the maximum clustering height used to define phases and is carried over at the same height in (**B**) and (**C**) to facilitate direct comparison of age-phase structure across sexes. **A** All individuals. Clustering resolves four distinct aging phases—early life (<35), early-midlife (35–44), late-midlife (45–64), and late life (≥65)—with AU-supported subclusters (e.g., <30, 35–44, 65–84). **B** Males. Despite the four-phase visual overlay, bootstrap support identifies two dominant clusters (<50 and ≥50), indicating a more compressed and binary age-phase structure in males. **C** Females. Bootstrap-supported splits yield three distinct molecular phases: early life (<40), midlife (40–64), and late life (≥65), with finer-grained AU-supported subclusters (e.g., <30, ≥75), reflecting more gradual and articulated age transitions.

To examine phase-structured dynamics (“epigenetic waves”) across the lifespan, we clustered age bins using SHAP profiles from our DNAm model. Notably, these four broad age strata were used only for correlation-based feature selection; SHAP-based phase discovery was performed on finer age groupings to enable higher-resolution identification of age-transition structure. This approach groups ages with similar patterns of CpG influence, thereby delineating intervals of coordinated remodeling and the transition windows between them. In Fig. 4A (all individuals), the dendrogram resolves four broad phases: an early-life wave (<35 years), an early-midlife reconfiguration wave (35–44), a late-midlife wave (45–64 years), and a late-life remodeling wave (65 + ). The youngest bins (<20, 20–24, 25–29) form a tight subcluster within the early-life wave, consistent with highly coordinated epigenetic control in youth. This grouping is supported by multiscale bootstrap validation, with AU-BP confirming significance at AU ≥ 95%. The 35–44 cluster is similarly supported, and 65+ separates cleanly, indicating a distinct late-life SHAP signature. A horizontal red dashed line in Fig. 4A marks the maximum clustering height used to define these four phases, and this threshold is conserved in (B and C) to allow direct visual comparison of phase boundaries across sexes. This cut height was selected based on the clustering structure observed in the full cohort (Fig. 4A) and applied uniformly to the sex-stratified dendrograms (Fig. 4B, C) to maintain interpretability across groups. To compare these SHAP-based phase boundaries with clustering performed directly on raw methylation data, we repeated the same hierarchical analysis using genome-wide β-values (Supplementary Fig. 5). While overall resolution was lower, the resulting dendrograms revealed broadly similar age groupings, supporting the robustness of the inferred aging phases.

Sex-stratified clustering revealed fewer statistically supported phases than the combined model. In males (Fig. 4B), while four nominal phases can be visually delineated by the conserved dashed line, only two macro-clusters are statistically supported by AU bootstrap values: a combined early-to-midlife cluster spanning <20–49 and a late-life cluster spanning 50–85+. Within these broader groupings, substructure is evident—for example, a 35–49 transitional bridge between youth and older ages, and a split within late life (50–69 vs. 70–85+), though these were not statistically supported by bootstrap resampling. The lower resolution in males may reflect more gradual CpG shifts across midlife, potentially due to the absence of a single inflection point—such as menopause in females—that drives synchronized methylation changes. In females (Fig. 4C), the dendrogram resolves three AU-supported age clusters: an early-life grouping (<20–29), a midlife cluster (40–64), and a late-life phase (65–85+). The 30–39 group bridges the early and midlife periods, with 35–39 marking a transitional node. Age bins within the 40–64 cluster (e.g., 40–54 vs. 55–59) are not statistically supported (AU < 95%), consistent with a more heterogeneous midlife state. Within the late-life cluster, the oldest age bins (75–79, 85+) begin to separate, suggesting increasing epigenetic heterogeneity with advanced age. Taken together, these results indicate that the number of statistically distinct aging phases differs by sex: four in the combined cohort (Fig. 4A), two in males (Fig. 4B), and three in females (Fig. 4C). These clustering patterns reflect differences in the structure and timing of CpG-influence shifts and provide a framework for further analysis of phase-specific molecular changes.

### Biological Processes Underlying Epigenetic Aging in Early Life

**Fig. 5:**
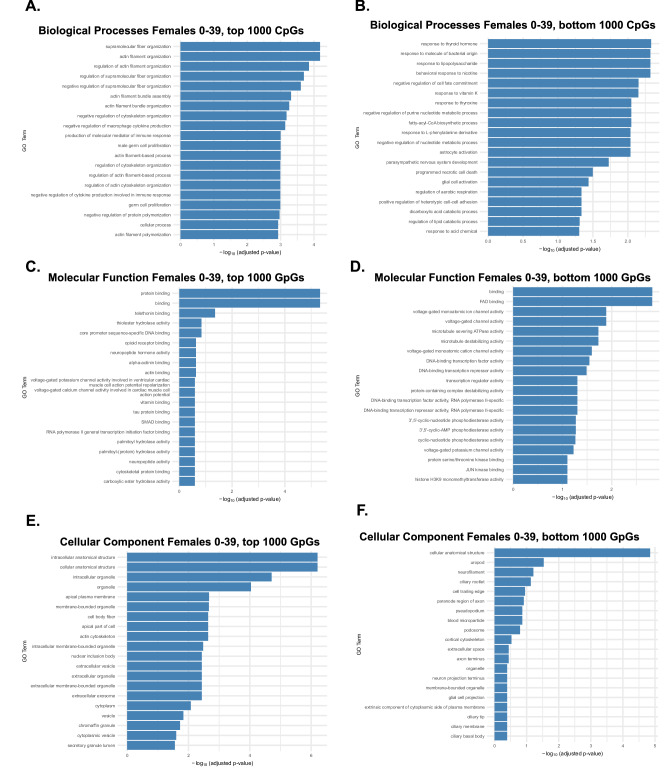
Female biological processes, molecular function, and cellular components associated with age-influential CpGs in early life (0–34 years). GO enrichment analysis was performed on the top 1000 CpGs most strongly correlated with age (high age-influence) and the bottom 1000 CpGs least correlated with age (low age-influence) in females during early life. Results are grouped by Biological Process (BP), Molecular Function (MF), and Cellular Component (CC). **A** BP enrichment of top CpGs revealed strong enrichment for actin filament organization, supramolecular fiber formation, and cytoskeletal remodeling pathways, indicating dynamic structural reorganization in early life. **B** BP enrichment of bottom CpGs showed enrichment for immune and hormonal regulatory processes, including thyroid hormone response, lipid metabolism, and epigenetic programming, suggesting these pathways exhibit more stable methylation during early life. **C** MF enrichment of top CpGs highlighted protein binding, ion channel binding, and voltage-gated channel regulation, while **D** broader MF enrichment included DNA-binding transcription factor activity and chromatin regulation, reflecting active developmental gene regulation. **E** CC enrichment of top CpGs mapped to intracellular anatomical structures, including the cytoskeleton, vesicles, and chromatin granules. **F** Bottom CpGs were enriched for membrane-associated compartments, such as the plasma membrane, synapse, and photoreceptor segments, suggesting relative stability in genes involved in structural signaling.

To investigate the biological relevance of age-associated CpG sites in early life (12.9–39 years), we performed GO enrichment analyses stratified by the top and bottom 1000 CpGs (by mean SHAP value) in females. This analysis revealed clear functional differences between CpGs that were strongly versus weakly associated with age. Biological Process (BP) enrichment of the top 1000 CpGs (Fig. 5A) revealed significant involvement of actin filament organization, supramolecular fiber assembly, and cytoskeletal remodeling pathways, along with processes, such as vitamin transport and stem cell proliferation, suggesting that early-life epigenetic aging in females predominantly targets genes regulating developmental and structural organization. In contrast, the bottom 1000 CpGs (Fig. 5B) were enriched for immune and hormonal regulatory pathways, indicating relative methylation stability in these systems during early life. Molecular Function (MF) analyses (Fig. 5C, D) supported this distinction. Highly age-influential CpGs were enriched for protein and ion channel binding, whereas lower-influence CpGs were linked to DNA binding and transcription factor activity, consistent with stable regulatory architecture during early development. Similarly, Cellular Component (CC) enrichment (Fig. 5E, F) showed that top CpGs localized to intracellular organelles, such as the cytoskeleton, vesicles, and chromatin granules, whereas bottom CpGs mapped to membrane-associated compartments including the plasma membrane and synapses. Together, these data suggest that early-life female epigenetic aging preferentially affects cytoskeletal and neurodevelopmental pathways, whereas CpGs associated with hormonal and metabolic regulation remain relatively stable.

Supplementary Fig. 2 extends these findings into mid-life females (40–64 years). Here, top age-influential CpGs were enriched for immune signaling, epigenetic regulation, and transcriptional activity, reflecting regulatory remodeling associated with perimenopause and endocrine aging. CpGs minimally correlated with age mapped to vascular and structural pathways, indicating continued stability in core tissue organization. Thus, mid-life female aging appears dominated by immune and transcriptional network remodeling, while preserving core structural processes.

**Fig. 6:**
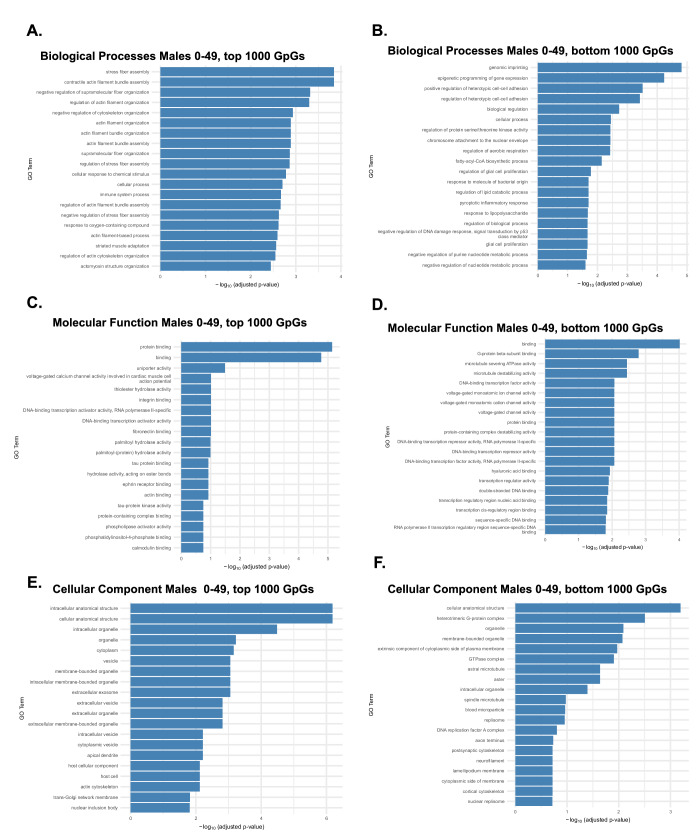
Male biological processes, molecular function, and cellular components associated with age-influential CpGs in early life (0–49 years). GO enrichment analysis was performed on the top 1000 CpGs most strongly correlated with age (high age-influence) and the bottom 1000 CpGs least correlated with age (low age-influence) in males during early life. Terms are grouped by Biological Process (BP), Molecular Function (MF), and Cellular Component (CC). **A** BP enrichment of top CpGs showed strong enrichment for stress fiber assembly, actin filament organization, cytoskeletal remodeling, and cell junction organization, highlighting structural and mechanical pathways as key targets of early male epigenetic aging. **B** BP enrichment of bottom CpGs revealed genomic imprinting, chromatin organization, purine metabolism, and cell proliferation pathways, suggesting these processes maintain greater methylation stability in early life. **C** MF enrichment of top CpGs highlighted protein binding, ion channel regulation, and hydrolase activity, while **D** broader MF enrichment included DNA-binding transcription factor activity, RNA polymerase II-specific regulation, and chromatin interaction, consistent with dynamic transcriptional regulation. **E** CC enrichment of top CpGs mapped to intracellular and membrane-bound organelles, including vesicles, endosomes, and mitochondria. **F** Bottom CpGs were enriched for nuclear and chromatin-associated compartments, such as the nucleoplasm, replication fork, and ribonucleoprotein complexes, indicating relative stability in genes critical for genome maintenance.

To explore the functional significance of age-associated CpGs in males aged 0–49 years, we performed analogous GO enrichment analyses. Distinct biological pathways again separated highly age-correlated CpGs from more stable sites. Top 1000 CpGs (Fig. 6A) were strongly enriched for cytoskeletal and structural organization, including actin filament bundle assembly, stress fiber formation, and axonogenesis, as well as muscle adaptation and cell junction organization, underscoring the early male emphasis on mechanical and structural cellular functions. Conversely, the bottom 1000 CpGs (Fig. 6B) were enriched for genomic imprinting, chromatin organization, purine metabolism, and DNA damage responses, suggesting that genes critical for genome maintenance and developmental regulation exhibit greater methylation stability in early male life. MF analyses (Fig. 6C, D) top-CpG enrichment for protein binding and ion channel activity, with bottom CpGs linked to transcriptional and chromatin-associated functions. CC analyses (Fig. 6E, F) again highlighted top CpGs localization to intracellular organelles, whereas bottom CpGs mapped predominantly to nuclear and chromatin compartments. Taken together, early-life male epigenetic aging appears primarily driven by changes in structural and signaling pathways, with relative preservation of genes tied to transcriptional and genomic stability, paralleling, but with subtle distinctions, the female early-life trajectory.

### Late-Life Epigenetic Aging Reveals Distinct Biological and Molecular Functions by Sex

**Fig. 7:**
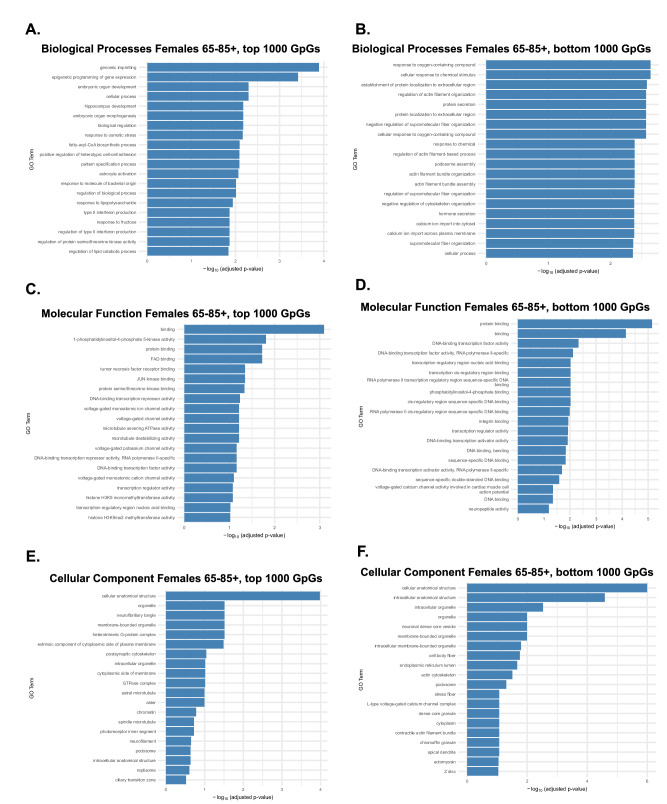
Female biological processes, molecular function, and cellular components associated with age-influential CpGs in late life (65–85 years). GO enrichment analysis was performed on the top 1000 CpGs most strongly correlated with age (high age-influence) and the bottom 1000 CpGs least correlated with age (low age-influence) in females during late life. Results are grouped by Biological Process (BP), Molecular Function (MF), and Cellular Component (CC). **A** BP enrichment of top CpGs revealed epigenetic and developmental pathways, including genomic imprinting, embryonic organ development, hippocampus development, and epigenetic programming of gene expression. Additional enrichment for immune and inflammatory signaling (e.g., type I interferon production, lipopolysaccharide response) indicates heightened immune regulation in late life. **B** BP enrichment of bottom CpGs showed cytoskeletal and cellular organization pathways, including actin filament bundle assembly, supramolecular fiber organization, and protein localization, suggesting stable methylation patterns in structural genes. **C** MF enrichment of top CpGs highlighted transcriptional regulation, particularly DNA-binding transcription factor activity and RNA polymerase II-specific sequence binding. **D** Broader MF enrichment of bottom CpGs also included DNA binding, chromatin regulation, and transcriptional activity, indicating these processes remain comparatively stable. **E** CC enrichment of top CpGs mapped to nuclear and chromatin-associated structures, such as the nucleoplasm, nuclear bodies, and protein–DNA complexes. **F** Bottom CpGs were enriched for intracellular and membrane-associated compartments, including the endoplasmic reticulum lumen, synapses, and photoreceptor segments.

To examine the biological functions associated with epigenetic aging in late-life females (65–85 years), we performed GO enrichment analyses on the top and bottom 1000 CpGs ranked by SHAP value. Compared to early life, which was dominated by cytoskeletal and structural pathways, late-life aging showed a pronounced shift toward regulatory and developmental programs. BP enrichment of the top 1000 CpGs (Fig. 7A) highlighted processes, such as genomic imprinting, epigenetic programming, hippocampus development, and immune signaling (e.g., type I interferon production), reflecting increased regulatory remodeling. The bottom 1000 CpGs (Fig. 7B) were enriched for cytoskeletal and localization pathways, reflecting structural stability over time. MF analysis (Fig. 7C, D) further supported this pattern, with top CpGs linked to transcription factor activity and RNA polymerase II binding, while bottom CpGs retained association with general protein binding and synaptic functions. CC enrichment (Fig. 7E, F) showed top CpGs localized to nuclear compartments, while bottom CpGs mapped to membrane-associated structures, such as synapses and the endoplasmic reticulum. Taken together, these findings suggest that late-life female epigenetic aging primarily targets transcriptional and developmental regulators, while genes involved in structural and synaptic maintenance remain comparatively stable.

**Fig. 8:**
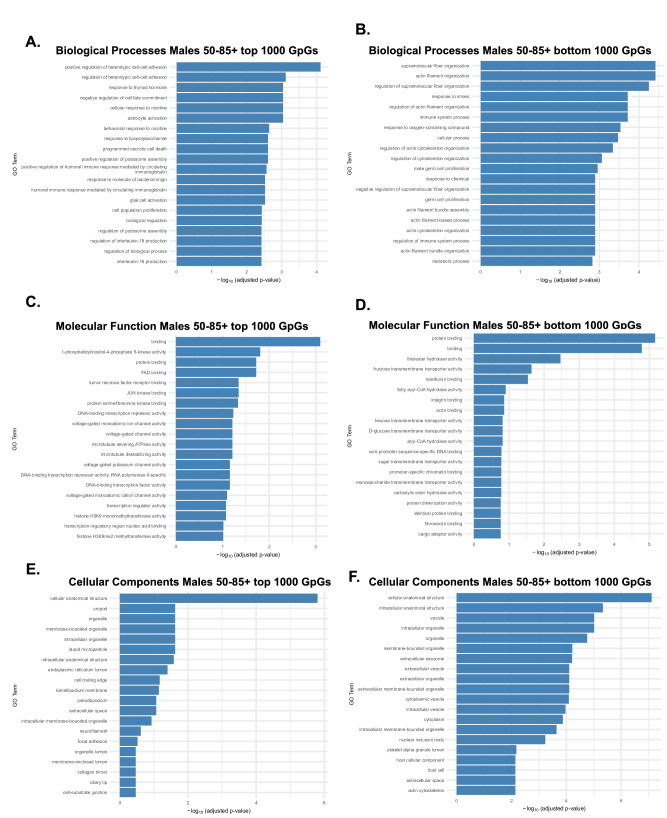
Male biological processes, molecular function, and cellular components associated with age-influential CpGs in late life (50-85 years). GO enrichment analysis was performed on the top 1000 CpGs most strongly correlated with age (high age-influence) and the bottom 1000 CpGs least correlated with age (low age-influence) in males during late life. Results are grouped by Biological Process (BP), Molecular Function (MF), and Cellular Component (CC). **A** BP enrichment of top CpGs revealed immune and inflammatory pathways, including regulation of heterotypic cell–cell adhesion, response to thyroid hormone, immunoglobulin-mediated signaling, and cytokine production (e.g., IL-1 and interferon-γ), highlighting immune regulation as a key target of late-life male epigenetic aging. **B** BP enrichment of bottom CpGs showed cytoskeletal and proliferative processes, including actin filament and supramolecular fiber organization, cell adhesion, and cell cycle regulation, suggesting that these structural pathways remain more epigenetically stable in late life. **C** MF enrichment of top CpGs highlighted protein binding, JUN kinase activity, histone modification (H3K4/H3K27 methyltransferase activity), and chromatin regulation, pointing to dynamic transcriptional and intracellular signaling changes. **D** MF enrichment of bottom CpGs included hydrolase activity, lipid binding, transporter activity, and promoter-specific chromatin binding, indicating relative preservation of these structural and metabolic functions. **E** CC enrichment of top CpGs mapped primarily to nuclear and intracellular compartments, including the endoplasmic reticulum, vesicles, mitochondria, and blood microparticles. **F** Bottom CpGs were enriched for membrane-bound organelles and extracellular vesicles, including synaptic vesicles, ribonucleoprotein complexes, and host cell components, suggesting selective stability in structural and secretory domains.

We next examined late-life (50–85 years) males. GO enrichment of the 1000 most and least age-influential CpGs revealed a shift toward immune regulation and cellular stress responses, alongside persistent enrichment of structural organization among CpGs with stable methylation. BP analysis (Fig. 8A) showed that the top 1000 CpGs were significantly enriched for immune and inflammatory pathways, including positive regulation of heterotypic cell–cell adhesion, response to thyroid hormone, immunoglobulin-mediated signaling, and cytokine production (e.g., IL-1 and interferon-γ). These enrichments suggest increased epigenetic remodeling of immune and inflammatory pathways in late-life males. Bottom CpGs (Fig. 8B) were enriched for cytoskeletal organization and cell cycle regulation, echoing early-life structural themes. These pathways echo those enriched in early-life CpGs in both sexes, suggesting that genes involved in structural maintenance retain greater methylation stability over time. MF analysis of the top CpGs (Fig. 8C) revealed enrichment for protein binding, JUN kinase activity, transcription factor binding, and histone modification activity (e.g., H3K4/H3K27 methyltransferase activity), indicating dynamic regulation of chromatin state and intracellular signaling as key features of male late-life aging. In contrast, bottom CpGs (Fig. 8D) were linked to lipid binding, transporter activity, and DNA binding, suggesting functional stability in structural and transport-related genes. CC analysis (Fig. 8E, F) emphasized similar compartmental patterns: top CpGs mapped to nuclear and intracellular structures, including the endoplasmic reticulum, vesicles, mitochondria, and blood microparticles, while bottom CpGs mapped to membrane-bound organelles and extracellular vesicles. In summary, late-life male epigenetic aging is marked by increased methylation changes in immune and transcriptional regulatory genes, whereas structural and transport-associated genes remain comparatively stable.

Finally, Supplementary Fig. 3 examines the clinical relevance of Δ-age (biological age minus chronological age), showing sex-stratified associations with adverse health outcomes after age adjustment. Supplementary Fig. 3A presents hazard ratios for time-to-event outcomes. While most outcomes showed no significant associations, Δ-age was linked to a higher hazard of cognitive decline, suggesting relevance for neurodegenerative risk. Supplementary Fig. 3B shows ORs for prevalent conditions at sample collection. After chronological age adjustment, Δ-age remained significantly associated with stroke, chronic kidney disease, cardiovascular disease (excluding stroke), and cognitive deficits, with consistent effects across both sexes. These associations were strongest for vascular and cognitive conditions, aligning with known impacts of accelerated epigenetic aging on the brain–vascular axis. The sex-stratified analysis revealed similar patterns for men and women, indicating that these relationships are robust across biological sex. Together, these findings indicate that Δ-age captures clinically meaningful variation in biological aging beyond chronological time.

## Discussion

In this study, we developed a deep learning–based epigenetic clock with stratified feature selection and SHAP-based interpretability, enabling accurate age prediction and sex-specific insight into aging trajectories. Across 29,167 lifespan-spanning methylomes, we identified three conserved epigenetic aging phases - early life, midlife, and late life - but their timing and structure were sex-specific. In males, early life (≤40 years) exhibited tightly clustered CpG signatures enriched for neural development and structural remodeling, followed by an abrupt midlife bifurcation (ages 45–49) characterized by immune and metabolic shifts. In contrast, females transitioned more gradually, with divergence beginning earlier (35–39) and progressing into a transcriptionally distinct late-life phase. In both sexes, age-informative CpGs showed dynamic regulatory shifts, while low-variability CpGs reflected stable structural programs across the lifespan. To visualize these transitions, we hierarchically clustered age groups by SHAP-derived CpG influence profiles (Fig. 3). A red dashed line in Panel A marks the maximum clustering height used to define four aging phases: early life (<35), early-midlife (35–44), late-midlife (45–64), and late life (≥65). This same threshold was applied to Panels B and C to enable direct comparison across sexes. While this four-phase structure is visually consistent, bootstrapping revealed sex-specific resolution: only two AU-supported phases were observed in males (<50, ≥50), indicating a more compressed trajectory, whereas females exhibited three statistically supported phases (<40, 40–64, ≥65), reflecting more gradual transitions. These patterns suggest that while men and women traverse shared epigenetic phases, they differ in the timing and pace of transitions.

Precisely modeling chronological age from DNAm is not orthogonal to outcome-based biological clocks but foundational to their interpretability. Chronological age remains the strongest correlate of morbidity and mortality; a model that approximates it with near-physical accuracy defines a normative aging manifold-against which deviations (Δ-age) can be meaningfully interpreted^8,9^. Our deep learning framework achieves this, offering state-of-the-art accuracy across cohorts and strong Δ-age associations with disease risk (e.g., cancer, stroke, CKD, CVD, cognitive decline). High-fidelity age prediction also reduces noise when estimating velocities (rates of epigenetic change) and curvatures (reconfiguration), supports rigorous change-point analyses, and supplies a clean substrate for pathway enrichment that links regulatory modules to specific aging phases^7,10–12^. These capacities transform clocks from descriptive biomarkers into tools for dynamical systems analysis. For example, hierarchical clustering of SHAP-derived “epigenetic waves” uncovered early-life stability, midlife reconfiguration, and late-life remodeling-paralleling the multi-omic waves described by Shen et al.^7^, who identified robust molecular crests in the 40 s and 60 s using a DE-SWAN^13^ sliding-window approach. While their study used longitudinal proteomics and metabolomics in 108 individuals, our approach leverages 29,167 cross-sectional methylomes and 12k CpGs to resolve the same crest pattern at CpG-level resolution with sex-specific precision. Methodologically, where DE-SWAN tests for molecular abundance shifts, our model infers the functional importance of CpGs to predicted age-linking life-stage inflections directly to regulatory programs and pathways. This alignment supports the idea that human aging follows coordinated, system-level inflection zones, and positions CpG-level SHAP analysis as a powerful lens for decoding the regulatory substrates of biological aging across the lifespan. Complementary immune-sensitivity analyses show that leukocyte composition accounts for a limited fraction of clock variance and that age correlations persist after adjustment (e.g., DeepStrataAge r = 0.846), indicating the SHAP-defined waves are not artifacts of cell-mix shifts but track cell-intrinsic regulation.

In addition to mapping structured aging phases, our modeling approach offers practical advantages in both accuracy and interpretability. It achieved an MAE as low as 1.89 years in our external validation, outperforming traditional linear and recent deep learning clocks. By selecting CpGs through age- and sex-stratified correlations, the model reduces demographic bias while capturing features relevant to specific life stages. Integration of SHAP^14^ enables individual-level attribution of CpG influence, supporting biologically meaningful interpretation. This framework bridges predictive performance with mechanistic insight, making it a versatile tool for both lifespan estimation and discovery. Beyond its technical strengths, the model enables detailed exploration of sex-specific molecular aging trajectories, beginning with the male profile.

Delving deeper into the male trajectory, early-life CpGs (≤40 years) the clustering pattern observed in Fig. 3B reflects two main phases, with early-life CpGs (≤40 years) forming a tightly grouped cluster consistent with a relatively stable epigenetic program during young adulthood. During this period, men maintain high testosterone levels, robust anabolic signaling, and balanced immune function, which likely stabilize methylation patterns linked to neural development, structural maintenance, and metabolic regulation. However, a sharp bifurcation emerged around ages 45–49, indicating an abrupt molecular transition in midlife. This aligns with andropause, the gradual yet biologically significant decline in testosterone, which disrupts endocrine regulation of muscle mass, adiposity, and immune function^15–17^. Lower androgen levels reduce anti-inflammatory signaling and vascular protection, leading to increased visceral fat deposition, metabolic dysregulation, and chronic low-grade inflammation-all of which drive divergence in the methylome. CpG enrichment in this phase was linked to immune and inflammatory pathways, late-life male CpGs (50–85 years) were strongly enriched for immune and inflammatory pathways, including cytokine regulation (IL-1, interferon-γ), cell adhesion, and immunoglobulin-mediated signaling (Figs. 6, 7). These signatures reflect immunosenescence-the decline in adaptive immune competence-and inflammaging, a chronic pro-inflammatory state fueled by senescent cells, altered gut microbiota, and oxidative stress^18–22^. This compressed midlife transition likely reflects the convergence of hormonal, metabolic, and immune shifts in men^23^.

Females, by contrast, showed earlier but more gradual SHAP divergence beginning around ages 35–39, extending smoothly through midlife and into late life. This trajectory mirrors the structured hormonal transition of perimenopause and menopause, which unfolds predictably over a decade-long window. Estrogen, a key regulator of immune modulation, vascular function, and neuroprotection, declines progressively, lifting anti-inflammatory constraints and gradually reshaping immune and stress-response networks^24^. Supporting this interpretation, midlife female CpGs (35–64 years) were enriched for immune signaling, stress response, and cytokine production, consistent with the well-documented shift toward low-grade pro-inflammatory states during estrogen decline and altered hypothalamic-pituitary-adrenal (HPA) function^25,26^. Unlike the abrupt transitions in males, female epigenetic remodeling appears phased and hormonally synchronized. By late life (65–85 years), female CpGs shifted toward transcriptional regulation, developmental pathways, and neuroendocrine signaling, reflecting a progressive shift in epigenetic trajectories influencing changing transcriptional networks. In the oldest female cluster (85+), SHAP profiles fragmented, indicating greater heterogeneity in biological aging among long-lived women, likely reflecting survival bias and divergent resilience phenotypes in extreme age.

Together, these results highlight shared molecular aging phases in men and women, but with distinct timing and transition dynamics. In men, midlife shifts appear abrupt and compressed, likely due to nonlinear endocrine decline, which unmasks latent immune and metabolic vulnerabilities. In women, transitions unfold earlier and more gradually, aligning with the neuroendocrine rhythm of menopause and promoting a phased remodeling of immune and transcriptional networks. Despite these differences, both sexes show stability in core methylation patterns, suggesting that aging mainly affects dynamic regulatory regions rather than foundational epigenetic structures.

Looking forward, several avenues emerge. First, experimental validation will be essential to confirm the functional relevance of the sex-specific CpGs and pathway enrichments identified in this study. Longitudinal studies will be critical to validate the temporal order of CpG transitions and disentangle cause from consequence in sex-specific aging. Expanding this framework to multi-tissue datasets could determine whether these inflection points represent systemic reprogramming or are blood-specific immune events. The identification of highly age-influential CpGs during midlife transitions also offers a roadmap for functional perturbation—e.g., via CRISPR/dCas9-based epigenetic editing—to test causal roles in immune or neuroendocrine remodeling^27^. Integrating these CpG signatures with transcriptomic, proteomic, and metabolomic data could further reveal upstream regulators orchestrating sex-specific aging phases. Finally, because we identify distinct windows of molecular transition—a gradual perimenopausal drift in women and a compressed bifurcation in midlife men—future interventions could time geroprotective strategies accordingly, from hormone replacement to immune-targeted therapies. We acknowledge several limitations, including the cross-sectional design of the study, the use of blood-derived methylation data, and the lack of batch correction, which may leave residual technical effects unaccounted for. Additionally, incomplete availability of race and ethnicity data limited our ability to assess the generalizability of findings across ancestral backgrounds. Third, although external and internal factors, such as smoking, alcohol consumption, psychosocial stress, race/ethnicity, menopausal status, and cardiovascular disease are known to influence DNAm profiles, these variables were not uniformly collected across the multicenter cohorts used for model training and therefore could not be explicitly adjusted for in the development of DeepStrataAge. Using an independent Harvard/MGB cohort with richer phenotyping, we performed sensitivity analyses adjusting for sex, age, smoking, and alcohol use and found that our main inferences involving DeepStrataAge predicted age and Δ-age were broadly consistent across multiple covariate sets (Supplementary Fig. 6A, B). We also note that age-stratified feature selection was based on a fixed set of broad age bins; future work should assess the sensitivity of findings to alternative binning strategies, such as narrower early-life bins or uniform decade bins, and evaluate the stability of selected CpGs and SHAP-based interpretations under different stratifications. Finally, while the present study establishes an interpretable and generalizable modeling framework, future work will focus on expanding validation to additional publicly available EPIC datasets and more diverse cohorts to further assess robustness and generalizability. Nevertheless, a more systematic evaluation of how these exposures shape DeepStrataAge estimates and model fairness across demographic and clinical subgroups remains an important direction for future work. Together, these findings not only refine our understanding of sex-specific aging, but also lay a translational foundation for precision geroscience.

## Methods

### Cohorts and Data Sources

We assembled whole-blood DNAm data from multiple cohorts to train and evaluate our deep learning–based epigenetic clock (Table 2). All samples were derived from peripheral whole blood, and within each cohort we curated metadata to retain a single sample per unique individual (no repeated measures were included). All data were generated using the Illumina Infinium MethylationEPIC v1.0 or MethylationEPIC v2.0 microarrays and included sample-level metadata, such as chronological age and sex. The dataset encompassed 29,167 samples in total. A large subset of samples was provided by TruDiagnostic (Lexington, KY), consisting of 22,603 individuals measured using both the original EPIC array and the updated EPIC v2.0 platform. Samples from TruDiagnostic were collected from April 2021 to approximately May 2024; Peripheral whole blood samples were obtained using the lancet and capillary method, as well as Dried Blood Spot (DBS), and preserved through mixing with lysis buffer. 500 ng of DNA was extracted and subjected to bisulfite conversion using the EZ DNAm Kit from Zymo Research, following the manufacturer’s protocols. The converted samples were then randomly allocated to designated wells on the Infinium HumanMethylationEPIC 850k BeadChip, and then subsequently amplified, hybridized onto the array, and stained. Samples were then washed and imaged using the Illumina iScan SQ instrument to capture raw image intensities. In addition, whole-blood profiles were collected from the Massachusetts General Brigham (MGB) Biobank, contributing 4743 individuals aged 13 to 96.4 years on the EPIC platform. The Biomarkers of Aging (BoA) cohort (GSE246337) provided 500 individuals aged 18.1 to 88.3 years, profiled on the EPIC v2.0 array. To increase the representation of healthy younger adults, we incorporated 570 individuals from the Center for International Blood and Marrow Transplant Research (CIBMTR) cohort (GSE196696), aged 18.6 to 39.3 years, profiled on the EPIC array. Finally, an independent set of 999 TruDiagnostic samples profiled on EPIC v2.0, aged 19.7 to 94.2 years, was reserved as a held-out validation cohort. To harmonize data across platforms, we retained those CpG sites that were present across both array platforms and all datasets (*N* = 689,541), which were used for downstream modeling and analyses. In all analyses, we restricted to the 689,541 CpG sites that were reliably measured on both the EPIC v1.0 and EPIC v2.0 arrays across cohorts to ensure cross-platform comparability.

**Table 2:** Cohorts, platforms, and sample characteristics used for model development and evaluation

Data source	Array Platform	Use	N	Median Age (Range)	Sex, n Male	Sex, n Female	Age and Sex Distribution	GEO
TruDiagnostic	EPIC + EPIC v2	Train + Test (LOCO)	22603	53.1 (12.9 - 99.8)	13403	9200		Internal
MGB	EPIC	Train + Test (LOCO)	4743	58.5 (13 - 96.4)	1620	2880		Internal
Biomarkers of Aging (BoA)	EPIC v2	Train + Test (LOCO)	500	52.8 (18.1 - 88.3)	239	256		GSE246337
CIBMTR	EPIC	Train + Test (LOCO)	570	27.8 (18.6 - 39.3)	423	147		GSE196696
TruDiagnostic	EPICv2	Validation	999	52.2 (19.7 - 94.2)	610	389		Internal

Rows list each data source, Illumina array platform (EPIC v1.0 and/or EPIC v2.0), and how the cohort was used: either for training and testing within a leave-one-cohort-out (LOCO) framework or as an external validation set. For each cohort we report the total sample size (N), median age with range (years), and sex counts (male/female). We also report the distribution of age stratified by sex, with males shown in light blue and females shown in light red, and vertical reference lines marking 0, 50, and 100 years. GEO entries include the accession number; “Internal” denotes non-public datasets. LOCO indicates that in each fold one cohort was held out entirely for testing while the remaining cohorts were used for training. Sex counts may not sum to N due to missing/unknown sex in the source metadata. Abbreviations: *EPIC* Illumina Infinium MethylationEPIC array (v1.0), *EPIC v2* Illumina MethylationEPIC v2.0, *GEO* Gene Expression Omnibus, *CIBMTR* Center for International Blood and Marrow Transplant Research, *MGB* Massachusetts General Brigham Biobank, TruDiagnostic, commercial laboratory cohort.

#### DNAm Processing

Genome-wide DNAm was profiled using the Illumina HumanMethylationEPIC platform (v1.0 and v2.0), with preprocessing conducted using standardized pipelines across internal and validation datasets. For all datasets processed from raw IDAT files, including TruDiagnostic (EPICv1 and EPICv2), MGB (EPICv2), and new BoA samples (EPICv2), we applied either the minfi or SeSAMe R packages using consistent parameters. For EPICv1 and EPICv2 TruDiagnostic samples used in model training, IDATs were imported using minfi, followed by single-sample Noob background subtraction, dye-bias correction, and β-value calculation with a + 100 denominator offset. Sample-level quality control was performed using the watermelon package and included thresholds for signal intensity (>5000), poorly performing probes (<5%), and abnormal beta distributions. EPICv2 TruDiagnostic validation samples were similarly processed in *minfi* using an updated manifest (20a1/hg38), with additional filtering based on detection p-values (mean >0.05) and ENmix QCinfo outlier detection; one sample flagged for high signal variability was excluded from downstream analysis. Address-to-probe collapsing was applied to yield one β-value per canonical CpG target. The final β-matrices were ssNoob-normalized and non-imputed.

For MGB samples, EPICv2 IDATs were processed using the SeSAMe package (v1.22.1) with default parameters, including background correction, dye-bias adjustment, and signal detection. Output was harmonized with EPICv1 probes by mapping loci and averaging multi-address probes. Public validation datasets were obtained fully processed. For GSE196696 (CIBMTR), we used SWAN-normalized β-matrices with all quality control and filtering performed by the original authors. For the BoA challenge dataset (GSE246337), previously released cohorts were used as provided. No additional normalization or probe filtering was applied to these public datasets beyond probe-ID harmonization for integration.

Batch correction was not applied, as methods, such as ComBat have been found to remove true age-related signal when age is not included as a protected covariate. Because age is unknown at inference time, applying ComBat would risk suppressing biological aging signal^28^. Instead, cohort-specific technical variation was addressed through a LOCO cross-validation framework, which evaluates model generalizability without relying on batch correction.

#### Feature Selection

To identify informative CpG sites for model training, we implemented a multi-step feature selection strategy based on age- and sex-stratified correlation analysis. Population counts for each age–sex subgroup are provided in Supplementary Table 1. Samples were grouped into four age bins (10–40, 40–50, 50–70, and 70–100 years) and stratified by sex within each bin. The age-bin boundaries were chosen to balance biological interpretability with empirical considerations, including preliminary model behavior and the age distribution of available samples. Because the cohort is skewed toward midlife, we used narrower bins in midlife and broader bins at the extremes to avoid unstable correlation estimates in sparsely sampled ranges. We did not perform a formal sensitivity analysis across alternative feature-selection binning schemes; evaluating how different stratifications affect CpG ranking stability and downstream interpretability is an important direction for future work. Within each age-sex subgroup, we calculated the Spearman correlation coefficient between methylation beta values and chronological age for all CpG sites. For each age group, the top 1% (*n* = 6895) of CpGs with the strongest absolute correlations were retained. CpGs selected across all subgroups were then merged to generate a non-redundant feature set, resulting in a total of 18,071 unique CpG sites. To ensure compatibility and consistent measurement across both the EPIC v1.0 and EPIC v2.0 arrays, we filtered the initial set of probes. This yielded a final feature set of 12,234 CpGs for use in our downstream modeling. We retained sex-chromosome CpGs because X-chromosomal markers have been shown to improve DNAm age prediction when integrated with autosomal CpGs, and excluding them a priori can discard informative age-associated signals^29^.

#### Deep Neural Network (DNN) Training

We trained a DNN to predict chronological age from DNAm profiles using a highly regularized architecture. This approach is consistent with recent studies demonstrating the effectiveness of regularized deep learning models for methylation-based age prediction^30^. The input feature set comprised the 12,334 CpGs together with a binary sex indicator to account for sex-associated methylation differences. For each dataset, samples were randomly split into an 80:20 ratio, with 80% used for training and hyperparameter tuning, and the remaining 20% reserved for independent testing.

Hyperparameter optimization was performed using the Optuna^31^ framework, which optimizes hyperparameters by iteratively testing and refining parameter combinations, using performance from earlier trials. In each Optuna trial, we trained a DNN under a LOCO scheme (k = 3), in which three cohort-specific models were independently trained, each excluding one of the external cohorts as shown in Fig. 1C, and evaluated on the corresponding held-out cohort. The hyperparameter search space included the number of hidden layers (*2–8*), hidden layer width (*128, 256, 512, or 1024 units*), dropout rate (*0.15–0.5*), L2 regularization strength (*10⁻⁶–0.1*), learning rate (*10⁻⁵–10⁻³*), and total number of training epochs (*10–5000*). The final model architecture and training parameters were selected based on those that yielded the lowest average MAE across all LOCO folds. Optuna identified an optimal configuration consisting of two hidden layers with 1024 units each, a dropout rate of 0.25, L2 regularization of 1.71 × 10⁻⁵, a learning rate of 3.06 × 10⁻⁵, and 5000 training epochs. Cohort-specific MAEs for all LOCO trials, as well as the aggregate performance used to select the optimal configuration, are provided in Supplementary Table 2. All model training was performed on a high-performance computing cluster using a single NVIDIA Tesla V100 GPU (32 GB VRAM) and 64 GB of system memory.

Information on lifestyle and clinical covariates (e.g., smoking, alcohol intake, psychosocial stress, menopausal status, and cardiovascular disease) was not available in a harmonized format across all cohorts used for model training. Consequently, these variables were not included as adjustment covariates during model development. Age and sex, which were consistently available, were used as core metadata, with sex incorporated into feature selection via age- and sex-stratified correlation analyses.

#### Epigenetic clock quantification

For the validation we quantified model outputs of both elastic net based and nonlinear chronological age clocks using the TruDiagnostic validation cohort. Canonical Horvath/Hannum/PhenoAge/DNAmTL were obtained via methylclock^32^. PC versions of Horvath1 (multi-tissue)^33^, Horvath2 (skin+blood)^33^, Hannum^33^, PhenoAge^33^, and DNAmTL^33^ used prefit objects (centers, rotations, coefficients, intercepts); required CpGs were checked and, if absent, imputed with whole-blood reference means (GSE40279), with any residual NAs filled by probe-wise cohort means before centering, projection, and back-transformation as specified.

Custom implementations covered Marioni cAge and RetroClock (v1/v2)^34^. Marioni cAge used elastic-net coefficients (linear and log-age sets) with per-CpG reference means to fill missing sites; linear predictions were computed for adults, while sub-20 predictions were recomputed with the log-age model and exponentiated. RetroClock estimates were the dot product of β-values and released coefficient tables plus the intercept, after imputing any missing CpGs from a reference panel.

AltumAge predictions were generated using the pyaging package in Python (https://github.com/rsinghlab/pyaging)^35^. GP-Age values were additionally calculated for each of the 10-, 30-, and 80-CpG models using the published implementation provided by the authors (https://github.com/mirivar/GP-age). All predictions were merged by Sample ID for validation. All other clocks in this analysis were calculated using Biolearn^36^.

### Associations to disease in the MGB Biobank

Δ-age is calculated as biological age minus chronological age. We calculated the association of Δ-age with all-cause mortality and 12 major diseases (cancer, COPD, asthma, cardiovascular disease - CVD, congestive heart failure, coronary artery disease, stroke, type 2 diabetes, chronic kidney disease, depression, cognitive deficit, chronic liver disease) in the MGBB test set. Diagnosis information was sourced from EMR data using ICD-9/10-CM codes. Prevalent and incident cases were identified by comparing the date of the first diagnosis with the date of plasma collection. We estimated the odds ratio (OR) using logistic regression and the hazard ratio (HR) using a Cox proportional hazards model. All models were adjusted for sex and chronological age to account for potential confounding factors. We also conducted sensitivity analyses to assess the influence of key covariates on downstream inferences involving DeepStrataAge. We compared unadjusted models using DeepStrataAge predicted age and Δ-age with eight covariate-adjusted specifications including sex, smoking status, alcohol use, and chronological age in different combinations (sex; sex + smoking; sex + alcohol; sex + smoking + alcohol; sex + age; sex + age + smoking; sex + age + alcohol; sex + age + smoking + alcohol). Full results are reported in Supplementary Fig. 6A, B.

#### Hierarchical clustering and GO

For inference, SHAP values were calculated using the GradientExplainer function from the SHAP package in Python. To examine age-related patterns, SHAP values were averaged within five-year bins spanning from individuals younger than 20 years to older than 85 years, resulting in 14 distinct age bins; sample counts for each bin are provided in Supplementary Table 3. Hierarchical clustering of these age-group profiles was performed using Ward’s method with the pvclust package in R, which computes AU-BP (approximately unbiased-bootstrap probability) support values and enabled the identification of clusters of age groups with similar SHAP distributions. This analysis was repeated for females and males. For each age group, the top and bottom 1000 CpGs were selected based on mean SHAP magnitude; this threshold was chosen after evaluating alternative set sizes (250, 500, 1000, 1500) and determining that 1000 provided the most stable enrichment results.

Functional annotation of these CpG sets was carried out using the GREAT (Genomic Regions Enrichment of Annotations Tool) framework as implemented in the rGREAT R package^37^, focusing on enrichment of GO terms to highlight biological processes, CCs, and molecular functions significantly associated with age-specific CpG subsets.

### Ethics Statement

Ethics approval and consent to participate: This study involved secondary analysis of previously collected, de-identified human DNAm array data obtained under appropriate data use agreements. No new human participant recruitment or biospecimen collection was performed for this work. The study involving human participants was reviewed and approved by the Institute for Regenerative and Cellular Medicine (IRB Approval Number IRCM-2022-336). All contributing studies/data sources obtained the relevant ethics approvals and were conducted in accordance with the principles of the Declaration of Helsinki and applicable local regulations.

## Supplementary information

Supplementary Information

Supplementary_Data_1_CpG_Comprehensive_Annotations

## Data Availability

The primary DNAm datasets generated and analyzed in this study include protected health information and are therefore not publicly available. De-identified, limited datasets (e.g., figure source data, aggregated summary statistics, model outputs, such as per-CpG SHAP summaries, and minimally necessary sample metadata) can be provided upon reasonable request to the corresponding author for non-commercial research purposes, subject to execution of a Data Use Agreement and verification of appropriate IRB/ethics approvals and institutional compliance (e.g., HIPAA/GDPR). Requests will be evaluated to ensure privacy, legal, and contractual obligations are met. Publicly available comparison cohorts used in this work can be accessed from GEO under accession GSE196696 and GSE246337. Any additional information required to evaluate the conclusions of the paper (e.g., detailed cohort inclusion/exclusion criteria and preprocessing parameters) is described in the Methods and Supplementary Information. All code for model training, evaluation, and clock inference has been deposited on Zenodo and is openly available at https://zenodo.org/records/17885641.
