# Supplementary material for: DeepStrataAge: an interpretable deep-learning clock that reveals stage- and sex-divergent DNA methylation aging dynamics
Source: NPJ Aging. 2026 Mar 13;12(1):62. doi: 10.1038/s41514-026-00358-w (PMC13128896; doi:10.1038/s41514-026-00358-w)
Supplement: Supplementary file 1 — Supplementary Information [file 41514_2026_358_MOESM1_ESM.pdf]

Supplementary Figure 1

A.

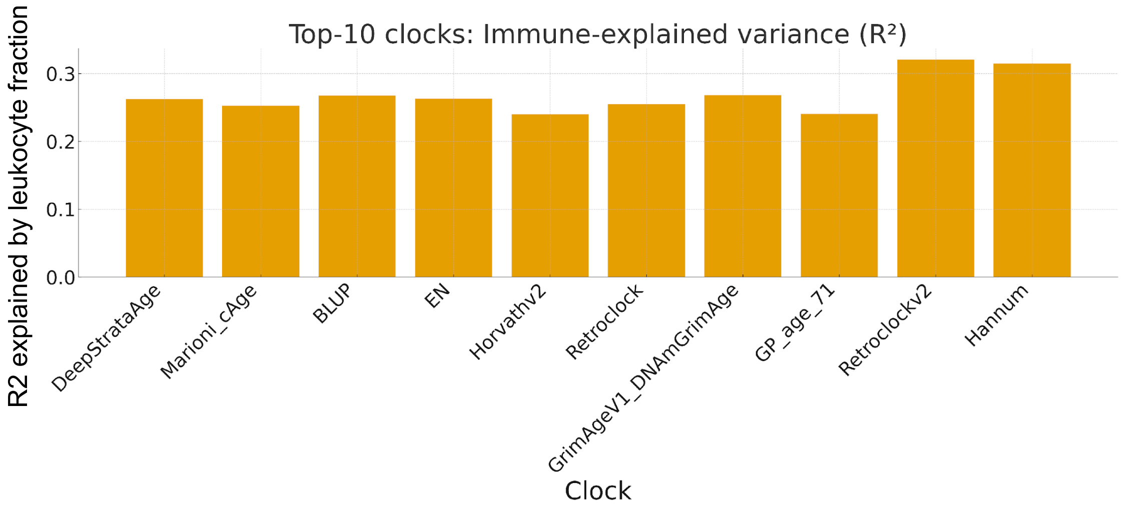

B.

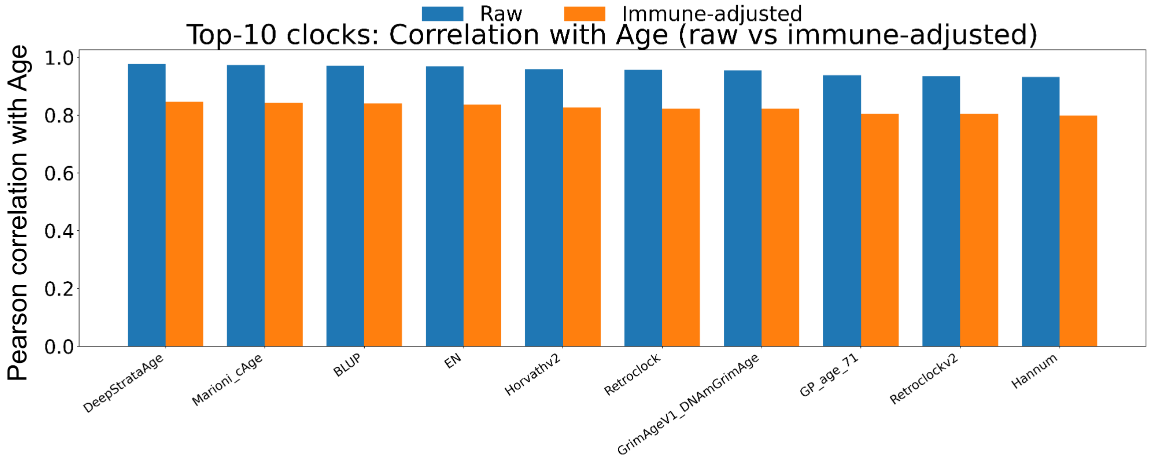

C. Correlation of immune cell subtypes to DeepStrataAge

Immune Cell	n	Pearson (r)	Pearson (p-value)	Spearman (rho)	Spearman (p-value)	Pearson (p-value, FDR)
**CD4Tmem**	999	0.1187	0.0002	0.1269	0.0001	0.0007
**Neu**	999	0.1094	0.0005	0.1029	0.0011	0.0016
**Baso**	999	0.0537	0.0896	0.0494	0.1189	0.1344
**Mono**	999	0.0517	0.1024	0.0626	0.0481	0.1366
**Bmem**	999	0.0337	0.2879	-0.0770	0.0149	0.3455
**Eos**	999	-0.0034	0.9149	0.0295	0.3509	0.9149
**NK**	999	-0.0174	0.5828	0.0031	0.9214	0.6358
**Treg**	999	-0.0828	0.0089	-0.0941	0.0029	0.0152
**CD8Tmem**	999	-0.0844	0.0076	-0.1081	0.0006	0.0152
**Bnv**	999	-0.0893	0.0047	-0.0975	0.0020	0.0113
**CD4Tnv**	999	-0.1702	0.0000	-0.1980	0.0000	0.0000
**CD8Tnv**	999	-0.4152	0.0000	-0.4134	0.0000	0.0000

Supplementary Figure 1. (A) Immune-explained variance (R^2). Bars show the fraction of variance in each epigenetic clock that is explained by leukocyte composition, estimated with the 12 Houseman/EpiDISH fractions (CD4Tnv, CD4Tmem, CD8Tnv, CD8Tmem, Treg, Bnv, Bmem, NK, Mono, Neu, Eos, Baso). For each clock, we fit an OLS model (clock ~ all cell fractions) and report the model R^2. The 10 clocks displayed are the top performers in this dataset by |Pearson r| with chronological age. n=999. (B) Correlation with age before and after immune adjustment. For the same clocks, blue bars give the raw Pearson correlation between the clock and chronological age; orange bars give the correlation after removing cell-composition effects by regressing the clock on the 12 leukocyte fractions and correlating the residual with age (i.e., “immune-adjusted”). This panel illustrates the magnitude of attenuation attributable to cell mix; ordering matches panel A. (C) Correlation of leukocyte fractions with DeepStrataAge. Table reports Pearson (r) and Spearman (ρ) correlations between DeepStrataAge and each leukocyte fraction (n=999). Two-sided P values are shown for both metrics; Pearson P values are additionally Benjamini–Hochberg FDR-adjusted. Consistent with immunosenescence, CD8 T-naïve (CD8Tnv) shows the strongest negative association with DeepStrataAge.

Supplementary Figure 2

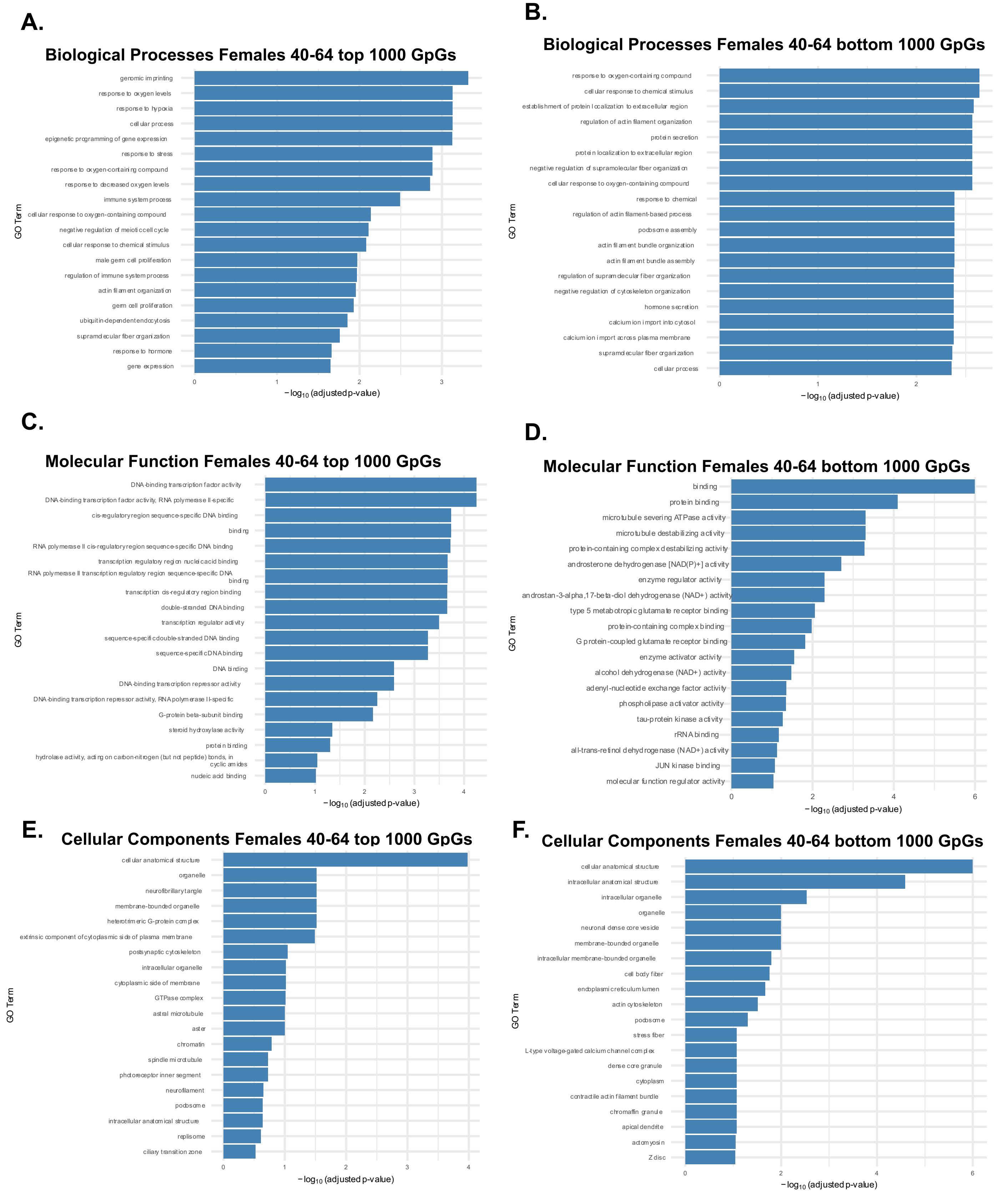

Supplementary Figure 2. Female biological processes, molecular function, and cellular components associated with age-influential CpGs in mid-life (35-64 years). GO enrichment analysis was performed on the top 1000 CpGs most strongly correlated with age (high age-influence) and the bottom 1000 CpGs least correlated with age (low age-influence) in females during mid-life. Results are grouped by Biological Process (BP), Molecular Function (MF), and Cellular Component (CC). (A) BP enrichment of top CpGs highlighted immune signaling, stress response, epigenetic programming, and cytokine production, consistent with regulatory transitions associated with perimenopause and endocrine aging. (B) BP enrichment of bottom CpGs showed vascular development, astrocyte activation, cell differentiation, and structural signaling pathways, indicating greater methylation stability in tissue organization during mid-life. (C) MF enrichment of top CpGs included protein binding, thiolester hydrolase activity, and promoter-specific chromatin binding, while (D) MF enrichment of bottom CpGs revealed metabolic and signaling-related activities, including kinase activity, ATPase binding, and growth factor receptor binding, suggesting these functions remain comparatively preserved. (E) CC enrichment of top CpGs mapped to intracellular and membrane-bound organelles, such as the nucleoplasm, vesicles, and cytoskeletal compartments. (F) Bottom CpGs were enriched for extracellular vesicles, blood microparticles, and membrane microdomains, reflecting relative stability in genes tied to extracellular communication and structural organization.

Supplementary Figure 3

A.

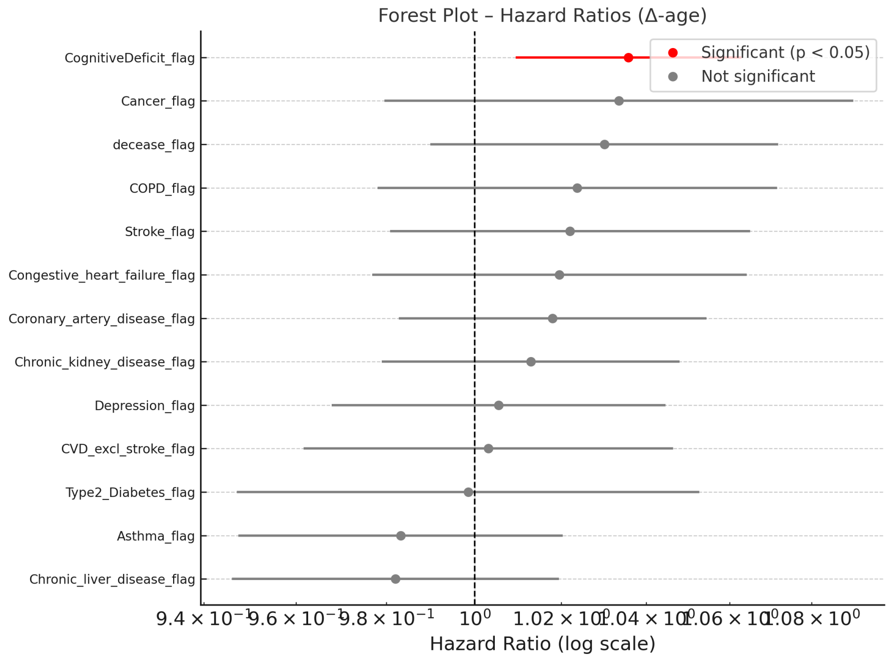

B.

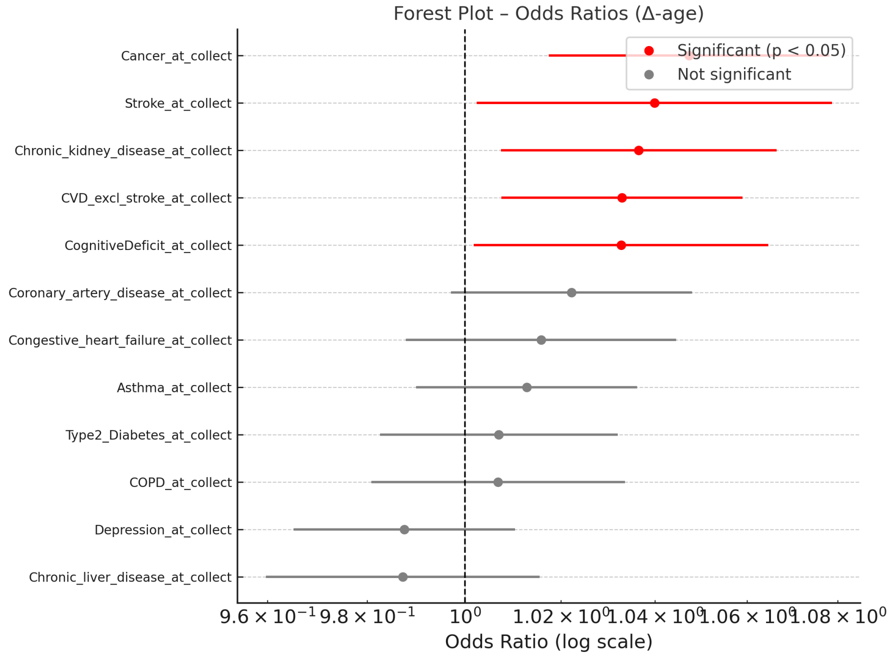

Supplementary Figure 3. Associations between Δ-age and clinical outcomes after adjusting for chronological age. (A) Forest plot showing hazard ratios for time-to-event outcomes in relation to Δ-age (biological age minus chronological age). Accelerated biological aging was significantly associated with increased hazard of cognitive decline, while other outcomes showed no significant associations after age adjustment. (B) Forest plot showing odds ratios for prevalent conditions at sample collection. Δ-age was significantly associated with higher odds of stroke, chronic kidney disease, cardiovascular disease (excluding stroke), and cognitive deficits, indicating that biological age acceleration captures cardiometabolic and neurovascular risk beyond chronological aging. Significant associations (AU ≥ 95%) are highlighted in red, while non-significant associations are shown in gray. Together, these findings suggest that Δ-age reflects clinically meaningful variation in biological aging, particularly affecting vascular and neurocognitive outcomes.

Supplementary Figure 4

A.

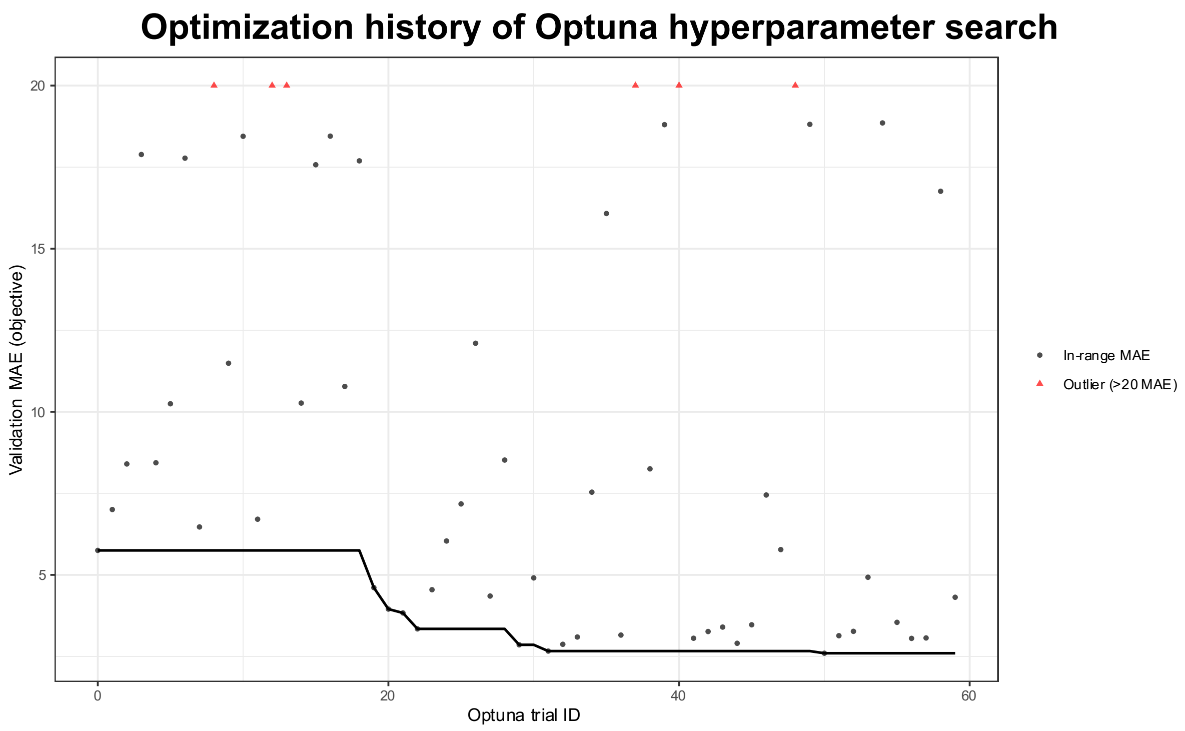

B.

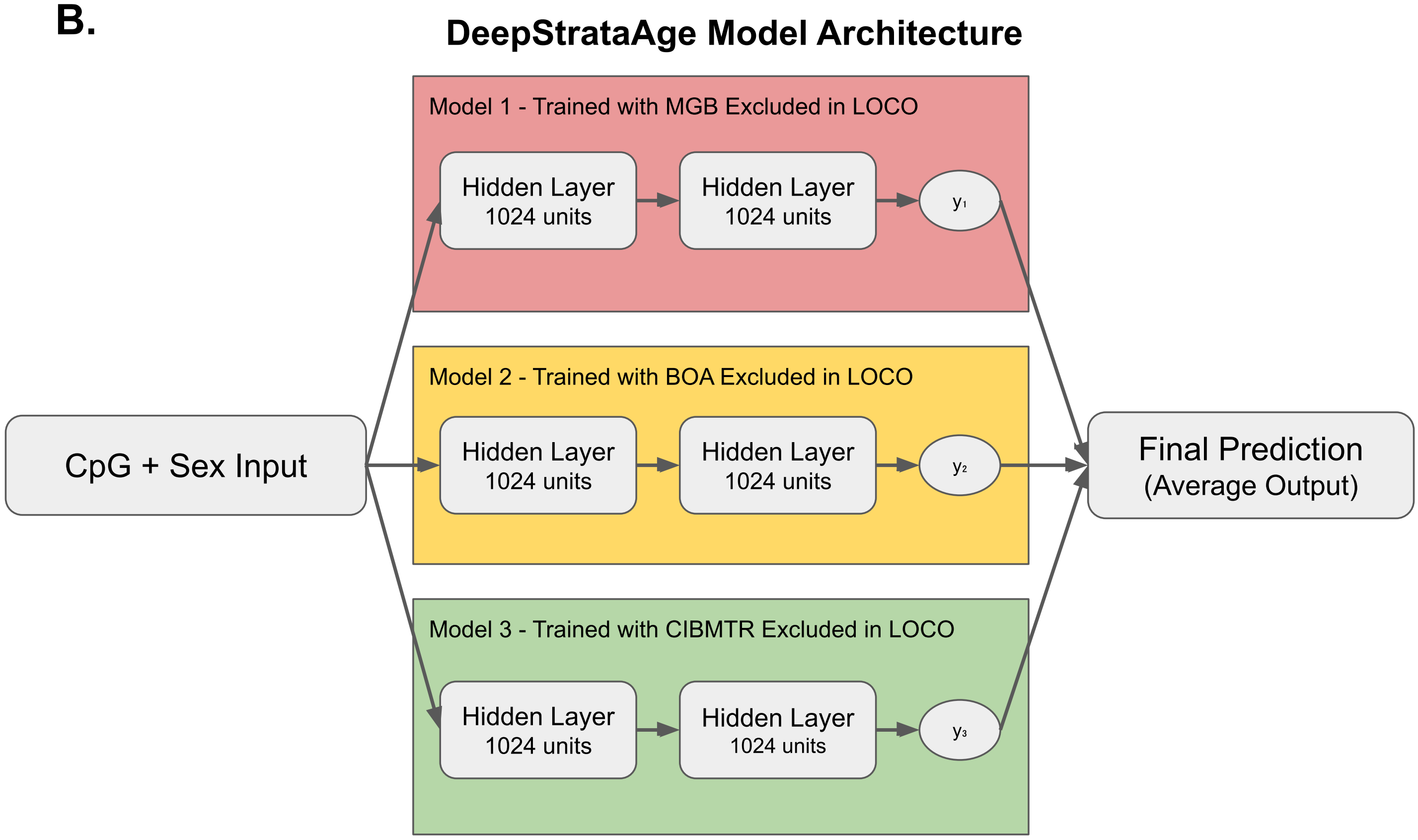

Supplementary Figure 4: Hyperparameter optimization and model architecture of DeepStrataAge. (A) Optimization history of the Optuna hyperparameter search used during model development. Each point represents a single trial with the corresponding mean absolute error (MAE) on validation data. Red triangles indicate outlier trials (MAE > 20). The black line tracks the lowest MAE achieved across trials, demonstrating convergence of the optimization process. (B) Schematic of the final DeepStrataAge model architecture. Three deep neural networks were trained using a leave-one-cohort-out (LOCO) framework, with MGB, BOA, and CIBMTR each held out in turn. Each network consisted of two fully connected hidden layers with 1024 units each. Final predictions were generated by averaging the outputs from all three models, forming an ensemble to enhance generalizability across cohorts.

Supplementary Figure 5

A.

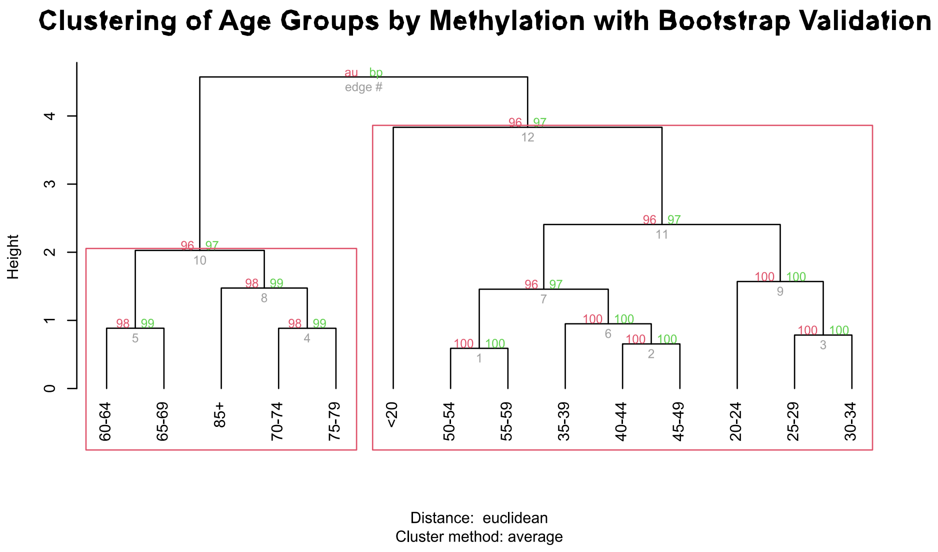

B.

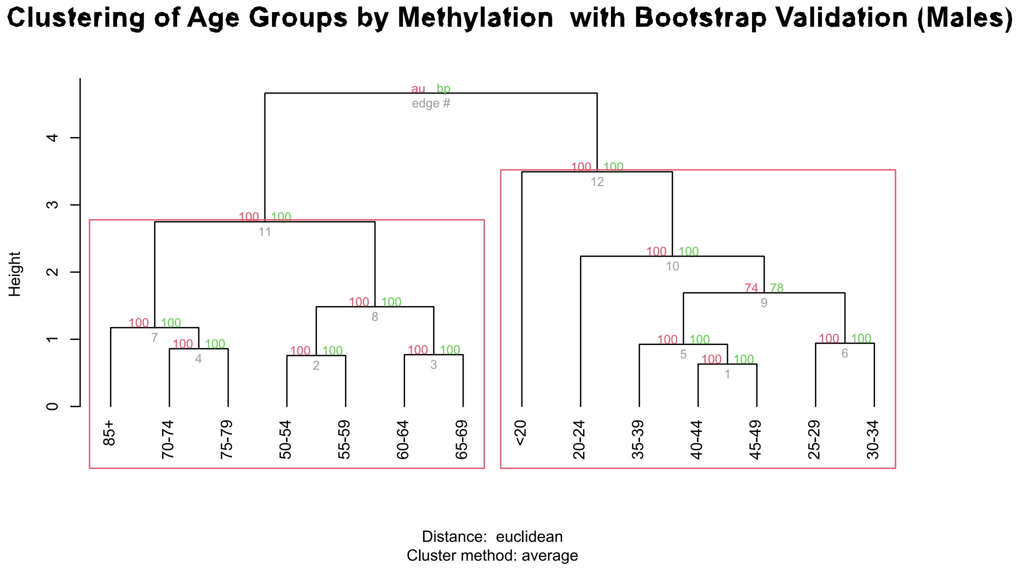

C.

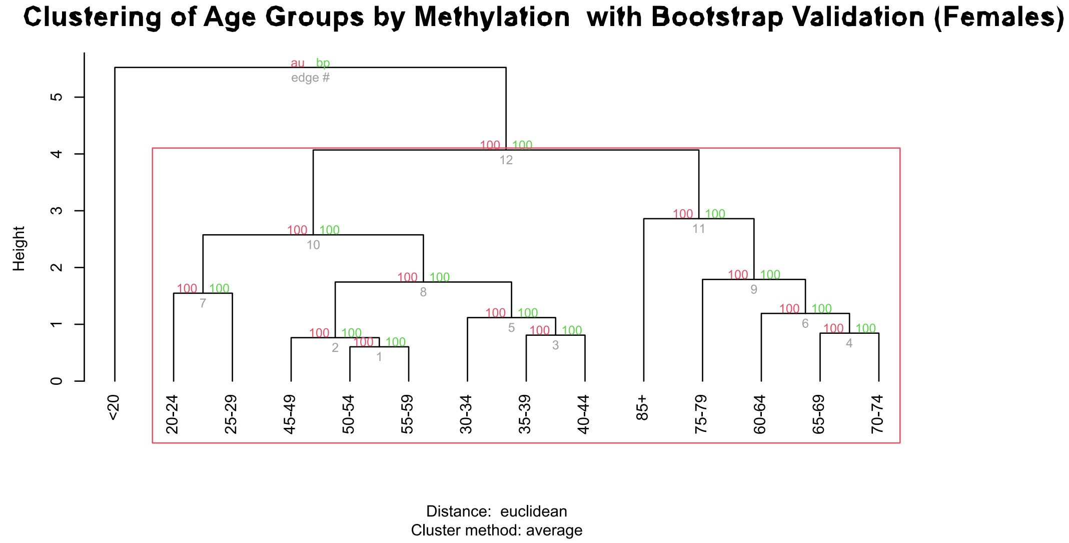

Supplementary Figure 5. Clustering of age groups by DNA methylation with bootstrap validation. Hierarchical clustering of 5-year age bins based on genome-wide methylation profiles, with multiscale bootstrap resampling (AU ≥ 95%) to assess cluster stability. Clustering was performed using Euclidean distance and average linkage. (A) Full cohort, showing four major branches separating early life, midlife, and late-life groups. (B) Male-only clustering reveals fewer statistically supported transitions, with broad early-to-midlife and late-life clusters. (C) Female-only clustering identifies more gradual phase separation, with clearer transitions between midlife and late life. These dendrograms highlight sex-specific differences in the timing and sharpness of methylation-based aging transitions.

Supplementary Figure 6

A. HR Beta Boxplots

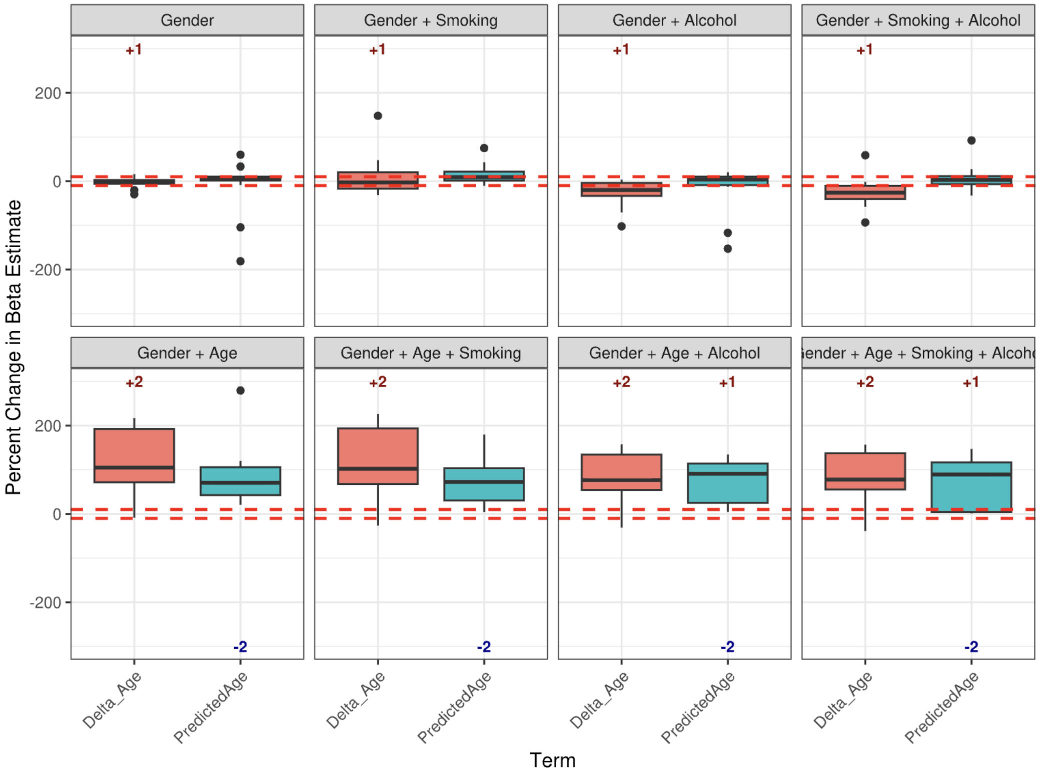

B. OR Beta Boxplots

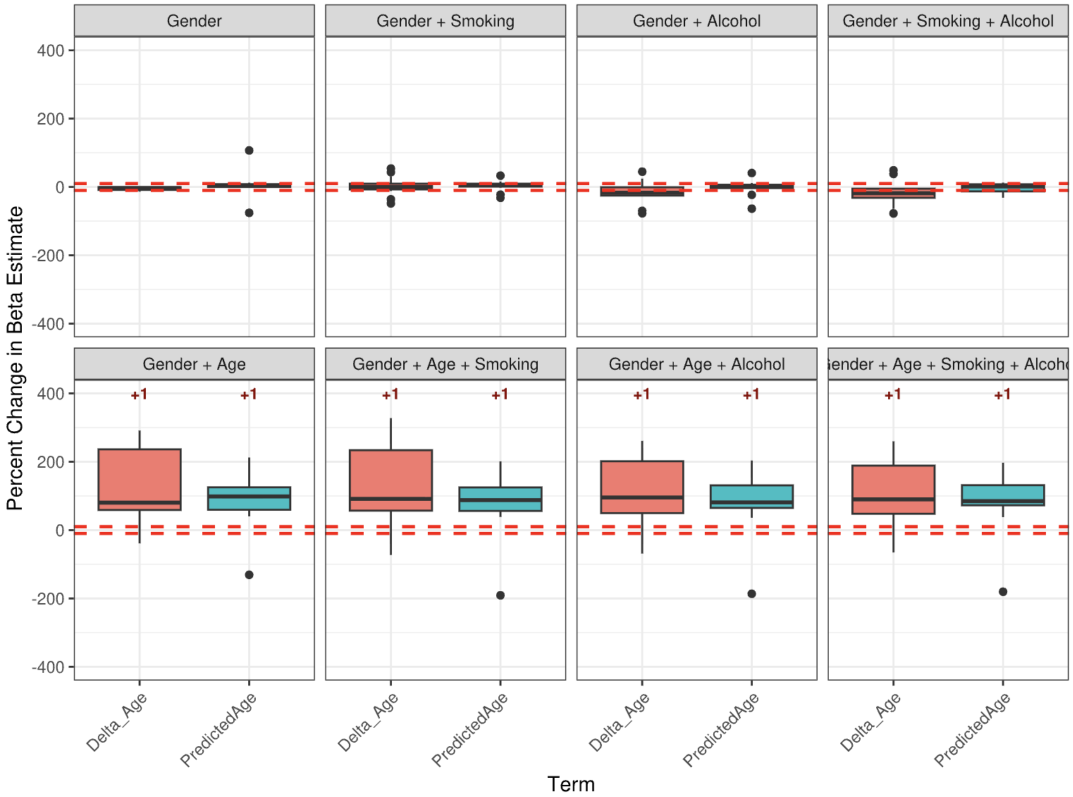

Supplementary Figure 6. Sensitivity analysis of covariate adjustment on model inference. Boxplots show the percent change in β coefficient estimates for DeepStrataAge and Δ-age across eight covariate adjustment models. Models included combinations of sex, smoking status, alcohol use, and chronological age. (A) Hazard ratio (HR) models for time-to-event outcomes. (B) Odds ratio (OR) models for prevalent conditions. Each panel represents a different covariate adjustment specification. Percent change in β estimates was computed relative to the unadjusted model. Results indicate that while adjustment for sex, lifestyle, and age covariates shifts effect sizes, the overall directionality and significance patterns of DeepStrataAge and Δ-age remain robust.

Supplementary Figure 7

A.

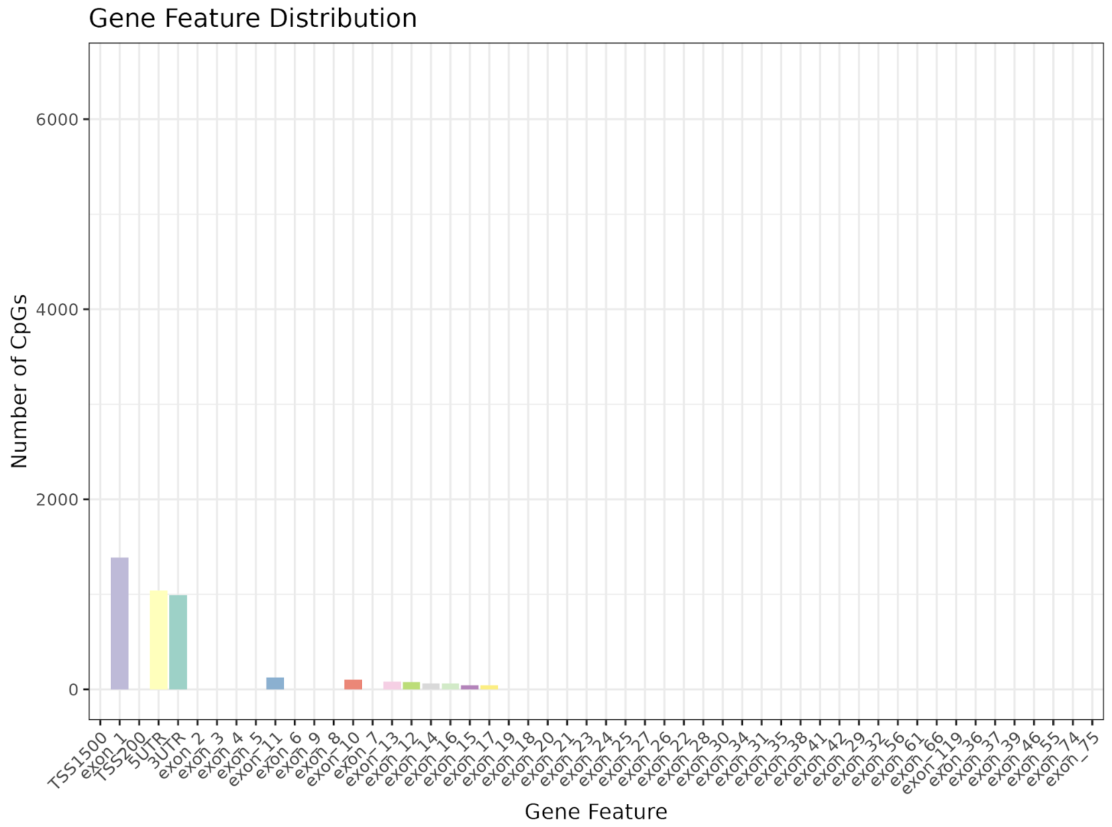

B.

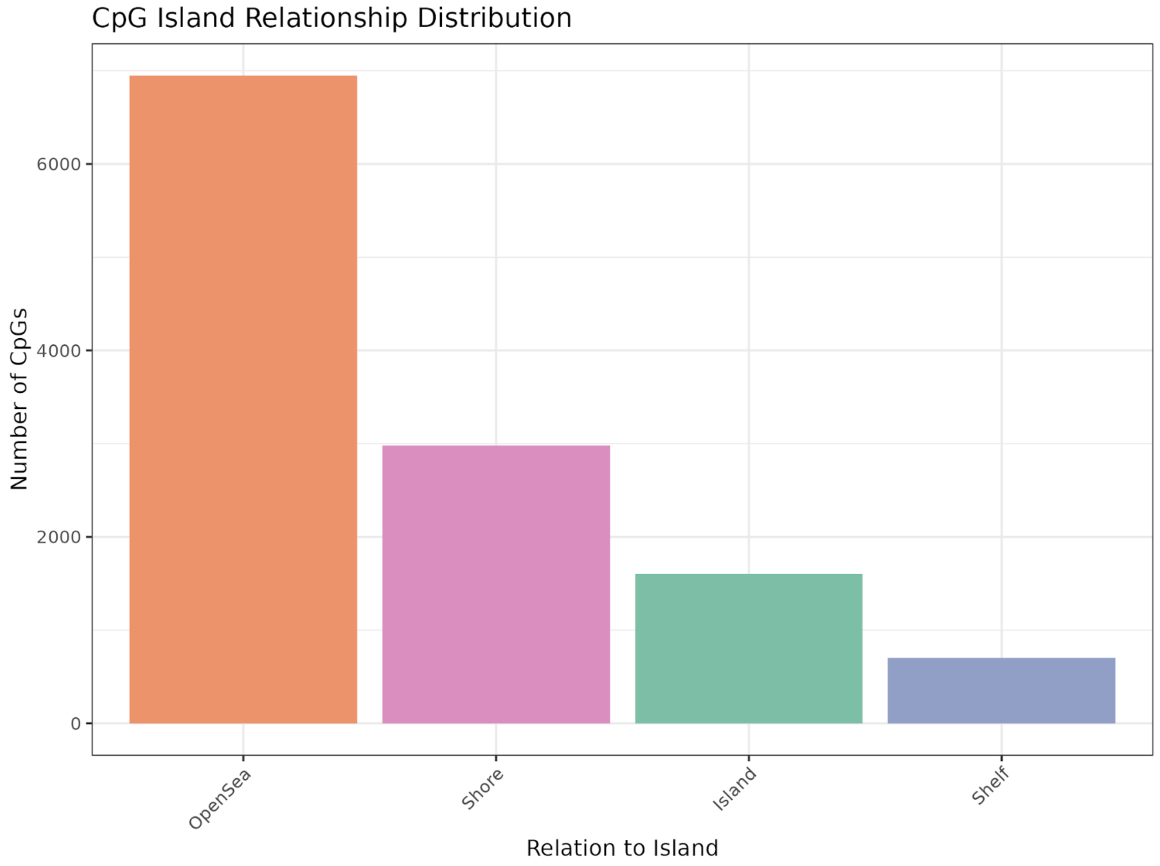

Supplementary Figure 7. Genomic annotations of the 12,234 CpGs used in DeepStrataAge. (A) Distribution of CpGs across annotated gene features (e.g., TSS1500, 5’UTR, exons, introns), based on the Illumina manifest. Most CpGs fall within promoter-associated regions (TSS1500, TSS200) and gene bodies. (B) Distribution of CpGs by CpG island context. The majority of CpGs are located in open sea and shore regions, with fewer in islands and shelves, consistent with enrichment in regulatory regions outside of CpG-dense promoters.

Supplementary Table 1. Cohort-by-age-by-sex sample counts used for stratified CpG feature selection.
Population counts for each age–sex subgroup used in the Spearman correlation–based feature selection procedure. Rows denote cohorts/datasets, and columns report the number of male and female samples within each predefined age bin (10–40, 40–50, 50–70, and ≥70 years; shown as 70–90 in the table based on the observed age range in these cohorts). Counts reflect the QC-retained samples contributing to subgroup-specific correlation estimates; zeros indicate that a given cohort contained no samples in that subgroup.

Cohort	10-40_Males	10-40_Females	40-50_Males	40-50_Females	50-70_Males	50-70_Females	70-90_Males	70-90_Females
TruDiagnostic	2303	1625	3155	2105	6512	4657	1423	801
MGB	172	605	202	469	780	1249	460	552
Biomarkers of Aging (BoA)	65	91	31	44	68	61	75	60
CIBMTR	423	147	0	0	0	0	0	0
TruD_Validation	100	79	165	123	278	159	65	28

Supplementary Table 2. Optuna hyperparameter optimization under leave-one-cohort-out (LOCO) cross-validation. Results from Optuna-driven hyperparameter tuning of the deep neural network (DNN) age model using a k = 3 LOCO scheme. Each row corresponds to a single Optuna trial and reports the tested hyperparameter configuration (number of hidden layers, hidden layer width, dropout rate, L2 regularization strength, learning rate, and training epochs), along with fold-specific mean absolute error (MAE; years) obtained when evaluating the model on each held-out cohort (cv_mgb_mae, cv_boa_mae, cv_cibmtr_mae). The optuna_objective_mae column reports the aggregate objective used for model selection (mean MAE across the three LOCO folds). The final model configuration was chosen as the trial with the lowest optuna_objective_mae.

trial_id	optuna_objective_mae	dropout	epochs	hidden_dim	l2_regularization	learning_rate	num_layers	cv_mgb_mae	cv_boa_mae	cv_cibmtr_mae
0	5.7497	0.25	1000	128	0.0000	0.0009	2	6.2470	9.2156	1.7865
1	7.0024	0.2	1000	128	0.0006	0.0001	5	8.6919	9.5815	2.7337
2	8.3997	0.4	5000	512	0.0000	0.0000	4	9.2929	10.4005	5.5058
3	17.8862	0.3	50	1024	0.0410	0.0007	2	14.2554	17.6880	21.7151
4	8.4359	0.15	1000	1024	0.0150	0.0000	8	9.1882	8.1860	7.9335
5	10.2443	0.35	1000	512	0.0002	0.0000	8	14.7643	11.7393	4.2295
6	17.7744	0.45	1000	256	0.0001	0.0008	4	23.1249	23.3648	6.8334
7	6.4680	0.35	1000	256	0.0001	0.0000	3	6.4184	7.1872	5.7985
8	41.6510	0.4	50	1024	0.0050	0.0000	7	53.3593	47.5367	24.0570
9	11.4877	0.15	500	512	0.0055	0.0001	8	14.7486	13.6183	6.0961
10	18.4450	0.25	100	128	0.0000	0.0003	2	15.5005	18.5745	21.2600
11	6.7056	0.3	1000	256	0.0000	0.0000	3	6.2354	5.8118	8.0696
12	42.1135	0.5	10	128	0.0000	0.0002	3	51.5055	50.7353	24.0996
13	41.0573	0.25	10	256	0.0003	0.0001	2	51.7933	48.8566	22.5220
14	10.2661	0.35	5000	256	0.0001	0.0005	3	16.9582	9.9934	3.8469
15	17.5722	0.25	500	128	0.0013	0.0000	6	20.0330	20.3126	12.3711
16	18.4489	0.4	100	128	0.0000	0.0001	4	19.6015	20.3481	15.3970
17	10.7761	0.2	1000	256	0.0001	0.0003	5	14.9895	12.8231	4.5157
18	17.6916	0.3	1000	128	0.0000	0.0000	3	16.4006	18.7784	17.8957
19	4.6078	0.35	1000	256	0.0000	0.0002	2	4.5865	3.9076	5.3294
20	3.9507	0.2	5000	256	0.0000	0.0002	2	3.9684	6.5031	1.3807
21	3.8320	0.2	5000	256	0.0000	0.0002	2	6.1568	3.5580	1.7812
22	3.3441	0.2	5000	256	0.0000	0.0002	2	3.2018	4.9175	1.9130
23	4.5444	0.2	5000	256	0.0000	0.0003	2	6.5009	4.1871	2.9452
24	6.0383	0.15	5000	256	0.0000	0.0001	4	8.4472	7.2321	2.4355
25	7.1744	0.2	5000	256	0.0000	0.0002	3	10.1717	8.7271	2.6245
26	12.1003	0.15	5000	256	0.0000	0.0005	6	16.7127	15.8566	3.7315
27	4.3508	0.2	5000	256	0.0000	0.0001	2	5.5736	5.2483	2.2304
28	8.5194	0.25	5000	256	0.0000	0.0002	3	12.1136	11.0394	2.4053
29	2.8560	0.2	5000	256	0.0000	0.0001	2	3.2275	2.6944	2.6461
30	4.9067	0.15	5000	1024	0.0000	0.0001	4	6.5943	6.0117	2.1142
31	2.6633	0.2	5000	256	0.0000	0.0001	2	3.3251	2.7189	1.9459
32	2.8715	0.25	5000	256	0.0000	0.0001	2	3.5845	2.7950	2.2348

33	3.0943	0.25	5000	256	0.0000	0.0001	2	3.3459	3.2609	2.6762
34	7.5352	0.25	5000	512	0.0000	0.0000	3	9.1174	8.5329	4.9551
35	16.0772	0.3	50	256	0.0008	0.0001	2	17.5936	19.2329	11.4052
36	3.1537	0.25	5000	256	0.0000	0.0000	2	3.3961	2.9331	3.1318
37	30.1914	0.3	10	1024	0.0000	0.0001	3	40.2012	36.8948	13.4780
38	8.2497	0.25	500	512	0.0001	0.0000	5	9.1346	8.3790	7.2355
39	18.8009	0.2	100	256	0.0000	0.0001	2	15.5552	19.1098	21.7378
40	28.4644	0.15	50	256	0.0001	0.0000	3	41.2156	32.3571	11.8206
41	3.0565	0.25	5000	256	0.0000	0.0001	2	3.2986	2.9307	2.9401
42	3.2631	0.25	5000	256	0.0812	0.0001	2	3.2423	2.8004	3.7466
43	3.3988	0.3	5000	256	0.0000	0.0001	2	5.3022	3.2199	1.6742
44	2.9018	0.25	5000	1024	0.0000	0.0000	2	3.2050	2.8273	2.6732
45	3.4692	0.2	5000	1024	0.0000	0.0000	3	3.9931	3.9200	2.4946
46	7.4477	0.3	5000	1024	0.0000	0.0000	4	8.1476	8.5024	5.6932
47	5.7745	0.25	500	1024	0.0004	0.0000	3	5.3994	5.4686	6.4555
48	36.4697	0.15	10	1024	0.0001	0.0001	7	47.1321	43.7414	18.5355
49	18.8106	0.45	100	512	0.0000	0.0000	2	15.5237	19.1280	21.7801
50	2.5972	0.25	5000	1024	0.0000	0.0000	2	3.1968	2.6586	1.9361
51	3.1345	0.25	5000	1024	0.0000	0.0000	2	3.2419	2.8838	3.2778
52	3.2685	0.25	5000	1024	0.0000	0.0000	2	3.3199	2.9978	3.4878
53	4.9258	0.2	5000	1024	0.0000	0.0001	3	6.3145	5.1765	3.2864
54	18.8538	0.3	50	1024	0.0001	0.0001	2	14.8775	19.1035	22.5806
55	3.5443	0.35	5000	128	0.0000	0.0000	2	4.3312	4.3244	1.9774
56	3.0523	0.2	5000	1024	0.0000	0.0000	2	3.4392	3.0213	2.6965
57	3.0660	0.2	5000	1024	0.0000	0.0000	3	3.6737	2.6449	2.8793
58	16.7595	0.2	500	1024	0.0000	0.0000	2	12.3756	15.6819	22.2210
59	4.3130	0.2	5000	1024	0.0017	0.0000	3	5.6459	4.0987	3.1945

Supplementary Table 3. Sample counts per SHAP age bin used for age-resolved interpretability analyses.
Counts of individuals included in each five-year age bin used to summarize model explanations from SHAP. For interpretability analyses, SHAP values were computed per sample and then averaged within age bins spanning from individuals <20 years through >85 years, yielding 14 age groups. Rows correspond to age bins and columns report the number of samples contributing to the SHAP-average profile for each bin; these bin-level profiles were subsequently used for hierarchical clustering (Ward's method) and bootstrap support estimation (AU/BP) in pvclust.

Age_Bin	Male_n	Female_n
<20	10	9
20-24	64	47
25-29	97	78
30-34	161	156
35-39	241	197
40-44	361	258
45-49	382	265
50-54	432	346
55-59	365	336
60-64	368	318
65-69	290	183
70-74	213	156
75-79	101	83
85+	76	41

Supplementary Table 4. CpGs significant by stratified Spearman correlation but not by unstratified Pearson correlation. List of CpG sites that emerged as significant age-associated loci in at least one age–sex subgroup using Spearman rank correlation, yet were not significant in the full, unstratified cohort under Pearson correlation. The table reports the CpG identifier (CpG) and the corresponding unstratified Pearson correlation coefficient (pearson_r) with its nominal p-value (p_value) and Benjamini–Hochberg FDR (FDR). These CpGs illustrate associations that are strengthened or revealed after stratification, consistent with non-linear and/or subgroup-specific age effects that can be diluted in aggregate linear analyses.

CpG	pearson_r	p_value	FDR
cg18302606	-0.01093177	0.071913256	0.072149134
cg11379439	0.010508871	0.083622724	0.08389013
cg20235075	-0.010481342	0.084435615	0.084698676
cg02888701	-0.010241813	0.091781367	0.092059766
cg13296579	0.010069111	0.097389702	0.097677105
cg00565348	0.010060907	0.097662809	0.097942989
cg01419713	-0.008325625	0.170497444	0.170972562
cg02926298	-0.008108766	0.18190723	0.182399194
cg16584406	0.007810501	0.198512124	0.199032683
cg23035488	0.007740608	0.202558654	0.203073184
cg23898189	-0.007693288	0.205332177	0.205836885
cg17371294	0.007346057	0.226531813	0.227070025
cg01862420	-0.007070862	0.244409402	0.24497002
cg02680932	-0.006684225	0.271164491	0.271764216
cg25432738	0.00656183	0.280037448	0.280633809
cg10243855	-0.006542445	0.281460711	0.282037003
cg16076654	-0.006176798	0.309225579	0.309833344
cg26805839	0.005773086	0.341915858	0.34255982
cg11199014	0.005719661	0.346401828	0.347025822
cg02555807	0.005446918	0.369883944	0.3705199
cg00732378	-0.005291004	0.383741461	0.384369773
cg02504956	-0.004811014	0.428357891	0.429024132
cg03142018	-0.004676973	0.441336533	0.441986779
cg18896834	0.004549289	0.453906498	0.454538059
cg04948438	-0.003449343	0.57014334	0.570889906
cg10149836	-0.003211208	0.597056748	0.597789632
cg02026141	0.002825332	0.641850105	0.642585389
cg03994108	0.002811795	0.643446808	0.644131214
cg08835992	-0.002753994	0.650282515	0.650920933
cg27120766	0.002625059	0.665636716	0.666235701
cg19382647	-0.00253226	0.676775657	0.677329257
cg01355392	0.001209093	0.842228498	0.842848492
cg02799972	0.001182136	0.845701452	0.846254786
cg00258809	-0.000991724	0.870313808	0.870812025
cg11513719	0.000837418	0.890352892	0.890789732
cg25384714	0.000787299	0.896877367	0.897244038

cg24407459	0.000724099	0.905114691	0.905410698
cg16204757	0.000641766	0.91586078	0.916085402
cg19499844	0.000556219	0.92704285	0.927194414
cg27114815	-0.000367377	0.951774579	0.951852377
cg10261093	0.000366056	0.951947808	0.951947808

Supplementary Table 5. Reference set of CpGs nonsignificant for age association under both unstratified Pearson and stratified Spearman analyses. CpG sites that did not exhibit significant age association under either (i) unstratified Pearson correlation in the full cohort or (ii) age–sex–stratified Spearman rank correlation within any subgroup. The table provides each CpG identifier (CpG) along with the corresponding unstratified Pearson statistics (pearson_r, p_value, and Benjamini–Hochberg FDR). This nonsignificant reference set delineates loci lacking detectable linear or monotonic age effects in the current data and provides a comparator to CpGs uniquely revealed by stratified rank-based analysis (Supplementary Table 4).

CpG	spearman_r	p_value	FDR
cg13296579	0.016729857	0.049738284	0.071608883
cg00732378	-0.006607912	0.43835051	0.499447734
cg15759827	0.003860392	0.650731058	0.701053855
cg07106394	0.013344327	0.117562569	0.155743349
cg00273464	0.011705782	0.169784617	0.216471083
cg13227833	0.012396196	0.145980539	0.189098998
cg15683950	0.012838037	0.132143787	0.172962511
cg13892386	-0.01388001	0.103541807	0.138949967
cg17371294	-0.009744733	0.253086196	0.308622491
cg17788468	0.007943941	0.351503597	0.411938748
cg18302606	-0.003878596	0.649194285	0.699631291
cg19499844	0.013799807	0.105552178	0.14138854
cg20235075	0.003893456	0.647940899	0.698511204
cg02799972	-0.003824542	0.653761862	0.703814564
cg11513719	-0.014363931	0.092049814	0.124996486
cg01312445	0.017840201	0.036397579	0.053930851
cg10149836	-0.00378354	0.657235252	0.706967664
cg20699340	-0.006497612	0.446032987	0.507027994
cg00021855	-0.014254166	0.094562276	0.128068543
cg02888701	-0.004541625	0.594280969	0.648931171
cg07090424	-0.002027012	0.812092563	0.844005736
cg21688248	0.008113768	0.341302448	0.401416813
cg00107782	0.014841757	0.081730761	0.112280645
cg15868152	-0.004506482	0.597137751	0.651585139
cg24387864	-0.003792179	0.65650278	0.706296173
cg03327263	0.012625576	0.138664863	0.180564257
cg22941637	0.009769699	0.251872328	0.307324345
cg07324822	0.003117257	0.714669913	0.758773425
cg27514197	0.008060497	0.344481689	0.404712523
cg04478883	-0.005504919	0.518525796	0.577355669
cg02283106	-0.00382178	0.653995599	0.70402644
cg10348972	-0.017572247	0.039300661	0.057827609
cg10261093	0.016143692	0.058298212	0.082702863
cg11308227	0.010339352	0.225272357	0.278432593
cg06741989	-0.017444939	0.040747433	0.059753428
cg01005308	-0.012463166	0.143814746	0.186591428

cg00152543	-0.003181576	0.709048116	0.753770052
cg00565348	0.015151162	0.075564312	0.104595691
cg15508761	0.012809377	0.133009313	0.173975637
cg27114815	0.012235992	0.151262668	0.195185728
cg04803798	0.002605472	0.759932697	0.798861041
cg24752967	0.000971371	0.909299633	0.92607079
cg13823643	-0.012515199	0.142149108	0.184633714
cg01355392	0.014927954	0.07997317	0.110093439
cg18896834	0.002451797	0.773694161	0.810843891
cg11379439	0.017753149	0.03732001	0.055175303
cg00356131	0.015666691	0.066136757	0.092710274
cg15522171	0.012767466	0.134282959	0.175466053
cg26091247	0.009775837	0.251574499	0.306996106
cg16204757	-0.000359274	0.966390677	0.972872817
cg02062196	-0.015574624	0.067745462	0.094757974
cg01419713	-0.01258205	0.140030915	0.182159725
cg11718501	0.013579424	0.111235899	0.148217896
cg00258809	0.002319451	0.785603066	0.821180398
cg04948438	-0.013036007	0.126283683	0.166069649
cg03306024	-0.010818557	0.204500643	0.255500305
cg11007075	0.012746161	0.134933981	0.176216619
cg01368737	0.013941943	0.10201033	0.137103787
cg25384714	-0.007798075	0.360418038	0.421084837
cg18270687	0.016993825	0.046242546	0.067027849
cg10298187	0.004998664	0.557710963	0.614692505
cg19382647	-0.011679146	0.170758056	0.217578174
cg10243855	-0.004467561	0.600308916	0.654494393
cg02026141	-0.012182251	0.153066812	0.197263937
cg14576825	0.011866241	0.16400819	0.209862686
cg03142018	0.003261136	0.702115987	0.747527229
cg20123374	0.004241043	0.618914037	0.671787335
cg26805839	-0.003978855	0.640757241	0.691981944
cg11697474	7.96E-05	0.992548037	0.994064255
cg27348423	-0.009720711	0.254258028	0.309886636
cg17931024	0.015580783	0.067636848	0.094617743
cg09825941	-0.009737946	0.253416908	0.308978445

Supplementary Data 1 (formerly Supplementary Table 6)

Comprehensive CpG annotation table. The complete CpG annotation table is provided as a separate spreadsheet file due to its size and to enable sorting/filtering by readers.

File: Supplementary_Data_1_CpG_Comprehensive_Annotations.xlsx (also provided as CSV).

Columns: CpG, ProbeID, chr, pos, strand, Islands_Name, Relation_to_Island, UCSC_RefGene_Name, UCSC_RefGene_Group, UCSC_RefGene_Accession, Regulatory_Feature_Name, Regulatory_Feature_Group, DMR, Enhancer.

Rows correspond to CpG probes included in the feature selection and modeling workflow; annotations are reported in hg38 coordinates and include gene context, CpG island context, regulatory feature labels, and flags for enhancer/DMR overlap when available.
